# Urokinase-type Plasminogen Activator Resulting from Endometrial Carcinogenesis Enhances Tumor Invasion and Correlates with Poor Outcome of Endometrial Carcinoma Patients

**DOI:** 10.1038/srep10680

**Published:** 2015-06-02

**Authors:** Chia-Yen Huang, Ming-Cheng Chang, Wei-Yun Huang, Ching-Ting Huang, Yu-Chien Tang, Hsien-Da Huang, Kuan-Ting Kuo, Chi-An Chen, Wen-Fang Cheng

**Affiliations:** 1 Gynecologic Cancer Center, Department of Obstetrics and Gynecology, Cathay General Hospital, Taipei, Taiwan; 2 Graduate Institute of Clinical Medicine, College of Medicine, National Taiwan University, Taipei, Taiwan; 3 Department of Obstetrics and Gynecology, College of Medicine, National Taiwan University, Taipei, Taiwan; 4 Department of Biological Science and Technology, Institute of Bioinformatics and Systems Biology, National Chiao Tung University, Hsin-Chu, Taiwan; 5 Graduate Institute of Oncology, College of Medicine, National Taiwan University, Taipei, Taiwan; 6 Department of Pathology, College of Medicine, National Taiwan University, Taipei, Taiwan

## Abstract

The purpose of this study was to identify the dysregulated genes involved in the tumorigenesis and progression of endometrial endometrioid adenocarcinoma (EEC), and their possible mechanisms. Endometrial specimens including normal endometrial tissues, atypical endometrial hyperplasia, and EEC were analyzed. The expression profiles were compared using GeneChip Array. The gene expression levels were determined by real-time RT-PCR in the training and testing sets to correlate the clinico-pathological parameters of EEC. Immunoblotting, *in vitro* cell migration and invasion assays were performed in human endometrial cancer cell lines and their transfectants. In microarray analysis, seven dysregulated genes were identified. Only the levels of urokinase-type plasminogen activator (uPA) were higher in EEC with deep myometrial invasion, positive lympho-vascular space invasion, lymph node metastasis, and advanced stages. After multivariate analysis, uPA was the only independent poor prognostic factor for disease-free survival in the EEC patients (hazard ratio: 4.65, p = 0.03). uPA may enhance the migratory and invasive capabilities of endometrial tumor cells by the phosphorylation of ERK1/2, Akt and p38 molecules. uPA is a dysregulated gene involved in the tumorigenesis, bio-pathological features and outcomes of EEC. uPA may be a potential molecule and target for the detection and treatment of EEC.

Endometrial cancer is the most common gynecologic cancer in Europe, with an estimated 98,920 new cases and 23,720 associated deaths in 2012^1^. In the United States, there were an estimated 47,130 new cases and 8,010 associated deaths in 2012^2^. Endometrial cancer is also the most common gynecologic cancer in Taiwan, with rapidly increasing incidence and mortality rates over the past several decades^3^. According to Bokhman’s categorization, there are two different pathogenetic types^4^. Type I carcinomas, accounting for 75-85% of cases, are prototypically of the endometrioid type, usually indolent in behavior and occurring in younger, peri-menopausal women with a background of endometrial hyperplasia with unopposed estrogen stimulation. In contrast, type II carcinomas are prototypically of the serous type, occurring in older, post-menopausal women with a background of atrophic endometrium, and associated with less favorable outcomes.

Endometrial endometrioid adenocarcinoma (EEC) is the most common histological type of endometrial cancer^5^. It is usually associated with chronic exposure to unopposed estrogen (either exogenous or endogenous) and is often preceded by atypical endometrial hyperplasia (AEH). Over the last 15 years, knowledge regarding the molecular genetics of EEC has increased substantially. Several studies have identified micro-satellite instability and mutations in the phosphatase and tensin homologue (*PTEN*) deleted on chromosome 10, *PIK3CA*, *K-ras, FGFR,* and *CTNNB1* (β-catenin) genes in cases of EEC^6^,^7^,^8^,^9^. Nevertheless, these molecular alterations are not present in all cases of EEC. For example, mutations and deletions of the *PTEN* gene occur in only 40% of EEC^10^.

For an accurate diagnosis and adequate treatment, the identification and understanding of the molecules responsible for cancer progression is critical. Because of the inconclusive findings and poor correlation between genotype and phenotype in EEC, the present study aimed to identify new potential genes that may be associated with cancer progression, invasion, or metastasis in EEC, and the possible mechanisms of these genes on EEC.

## Results

### Clinicopathological data of the 169 women with EEC

The basic clinicopathological parameters of the 169 EEC patients are shown in Table 1. Age, FIGO stage, tumor grade, depth of myometrial invasion, lymphovascular space invasion (LVSI), and lymph node metastasis were not different between the training set and testing set. The majority of the EEC patients were diagnosed as stage I (70%). In terms of histological grade, 112 patients (66%) had grade 1 tumors, 24 (14%) had grade 2, and 33 (20%) had grade 3. Of the 169 tumors, 67 (40%) were positive for LVSI, and lymph node metastasis was detected in 34 (20%).

### Candidate genes selected by comparative micro-array analysis and hierarchical clustering

Data from the four pooled samples (NEM, AEH, early-stage EEC, and advanced-stage EEC) that passed the quality control parameters were accessed through the National Center for Biotechnology Information via the Gene Expression Omnibus data repository (http://www.ncbi.nih.gov/geo/) accession numbers GSM956132–GSM956135. The Human Genome U133A Plus 2.0 array represented 54613 probe sets. To examine individual transcripts within the expression patterns and determine which genes were significantly and differentially expressed between the four samples, differentially expressed genes were chosen based on a high-fold change (at least four-fold change in at least one group comparison). This resulted in the identification of 41 genes that differed significantly among the four groups (Table 2).

### Confirmation of the comparative micro-array results by SQ RT-PCR

**Figure 1:**
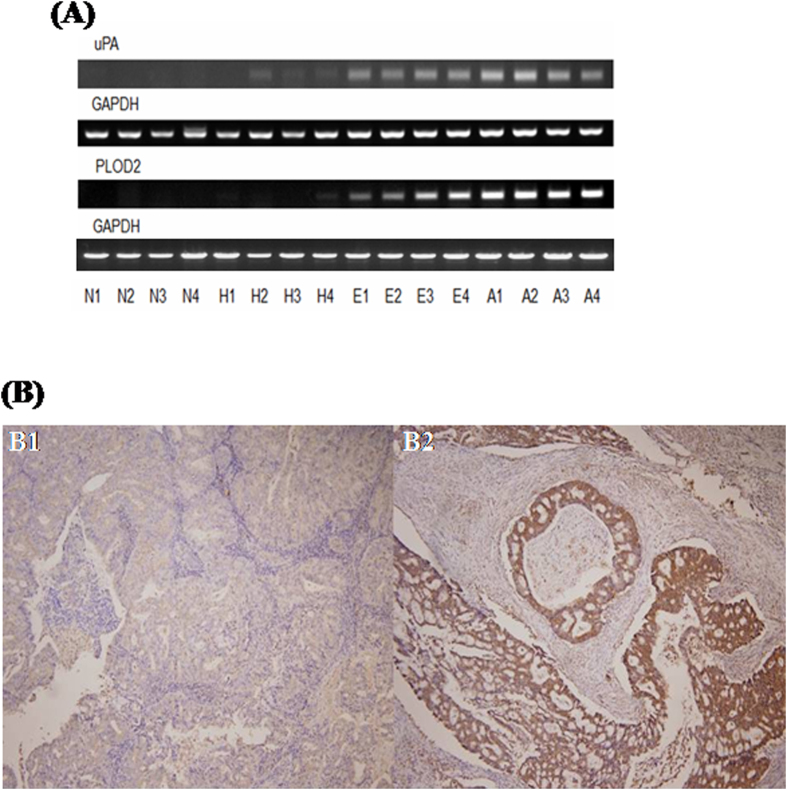
(**A**) Representative figures of uPA, PLOD2, and GAPDH mRNA expressions in different pathological conditions of endometrial tissues. N1 ~ N4, normal endometrium; H1 ~ H4: atypical hyperplastic endometrium; E1 ~ E4, early stage endometrial adenocarcinomas; A1 ~ A4: advanced endometrial adenocarcinomas by SQ RT-PCR. (**B**) Representative figures of negative staining (-) (B1, left panel) (grade 1 tumor with superficial myometrial invasion and without lymph node metastasis) and (**B**) strongly-positive staining (+++) (B2, right panel) (grade 2 tumor with deep myometrial invasion and positive pelvic lymph node metastasis) of uPA in endometrial cancerous tissues by immunohistochemical staining. *Note:* The slide was counter-stained with Mayer’s hematoxylin (10 × 40). uPA was mainly stained in the cytoplasm of endometrial cancer cells.

The selected 41 candidate genes were further screened and confirmed by SQ RT-PCR. The representative figures of SQ RT-PCR for uPA and PLOD2 are shown in Fig. 1A. Only when the expression of a candidate gene was higher in the EEC group than in the NEM and AEH groups the gene was considered significant. Of the 41 candidate genes, only seven (COL1A2, uPA, PSAT1, SLC2A1, GCLM, TTYH2, and PLOD2) had the same expression patterns as uPA and PLOD2. Thus, these seven genes were selected for further validation.

### Correlation between mean mRNA expression levels of the seven genes by RTQ RT-PCR in the training set

The expression levels of the seven respective genes selected by SQ RT-PCR were then evaluated in the training set that included 10 NEM, 10 AEH, and 85 EEC samples by RTQ RT-PCR. Correlations between clinicopathological parameters and expression levels of the seven genes were then evaluated in the training set. The relative expression levels of uPA (uPA/GAPDH x10^−3^) and PLOD2 (PLOD2/GAPDH x10^−3^) showed a significantly increasing trend from NEM, AEH to EEC (for uPA: 2.82 (NEM), 9.04 (AEH), 15.01 (EEC) *p* < 0.01; for PLOD2: 1.50 (NEM), 2.70 (AEH), 4.23 (EEC), *p* = 0.024; by one-way ANOVA) (Table 3). There was no increasing trend in the other five genes (i.e., COL1A2, PSAT1, SLC2A1, GCLM, and TTYH2).

Tumors with deep myometrial invasion (>1/2 myometrial depth) (19.4 vs. 12.9, *p* = 0.028), *p*ositive LVSI (17.2 vs. 13.0, *p* = 0.042), lymph node metastasis (34.1 vs. 12.1, *p* < 0.01), and advanced FIGO stages (stage III/IV) (25.7 vs. 12.7, *p* = 0.017) (all by one-way ANOVA) had significantly higher expression levels of uPA compared to those with superficial myometrial invasion (<1/2 myometrial invasion), negative LVSI, no lymph node metastasis, and early FIGO stages (stage I/II) in the training set (Table 3). There was no significant difference in uPA expression among the different histological tumor grades (G1, 15.0 vs. G2, 15.1 vs. G3, 14.5, *p* = 0.99, by one-way ANOVA).

The expression levels of PLOD2 were also significantly higher in the tumors with deep myometrial invasion (>1/2 myometrial depth) (5.14 vs. 3.79, *p* = 0.041, one-way ANOVA) and higher histological grade (G3) (G1, 3.46 vs. G2, 5.28 vs. G3, 6.25, *p* = 0.039, one-way ANOVA) compared to those with superficial myometrial invasion (<1/2 myometrial invasion) and lower histological grades (G1 and G2) among the patients with endometrial adenocarcinoma (Table 3). Nevertheless, there was no significant difference in PLOD2 expression in the other clinicopathological parameters including FIGO stage, LVSI status, and with or without lymph node metastasis.

There were no significant differences in the expressions other five genes, COL1A2, PSAT1, SLC2A1, GCLM, and TTYH2 in terms depth of myometrial invasion, LVSI status, tumor histological grade, status of lymph node metastasis, and FIGO stage.

### Validation of the mRNA expression levels of uPA and PLOD2 with various clinicopathological parameters in the testing set

To validate the findings from the training set, the mRNA expression levels of uPA and PLOD2 were further evaluated in another 10 NEM, 10 AEH, and 84 EEC samples of the testing set. Age, FIGO stage, tumor grade, and other clinicopathological parameters were comparable between the training and testing sets. The relative expression levels of both uPA (uPA/GAPDH x10^−3^) and PLOD2 (PLOD2/GAPDH x10^−3^) were also significantly higher in the EEC group compared to the AEH and NEM groups (for uPA: 2.68 (NEM), 8.67 (AEH), 13.72 (EEC) *p* < 0.01; for PLOD2: 1.73 (NEM), 2.58 (AEH), 4.69 (EEC), *p* = 0.012; by one-way ANOVA) (Table 4).

The tumors with deep myometrial invasion (>1/2 myometrial depth) (19.4 vs. 10.1, *p* = 0.025), *p*ositive LVSI (18.0 vs. 10.9, *p* = 0.038), lymph node metastasis (20.9 vs. 11.5, *p* = 0.018), and advanced FIGO stages (stage III/IV) (20.9 vs. 11.5, *p* = 0.018) (all by one-way ANOVA) had significantly higher uPA expressions compared to those with superficial myometrial invasion (<1/2 myometrial invasion), negative LVSI, no lymph node metastasis, and early FIGO stages (stages I/II) (Table 2).

However, the expression levels of PLOD2 in the testing set differed from those in the training set, and did not show significant differences in depth of myometrial invasion and tumor histological grade (Table 2).

### Correlations between protein and mRNA expression levels of uPA by IHC and RTQ RT-PCR

To further evaluate the expression levels between mRNA and protein in the EEC samples, IHC was performed to determine the protein expression patterns of uPA and correlate these with the results of RTQ RT-PCR. The representative figures of IHC for uPA are shown in Fig. 1B1 (negative) and 1B2 (strongly-positive). Of the 86 samples, 64 (74.4%) were positively (including weakly, moderately, and strongly) stained for uPA. This staining was mainly localized in the tumor cell cytoplasm (Fig. 1B2).

**Figure 2:**
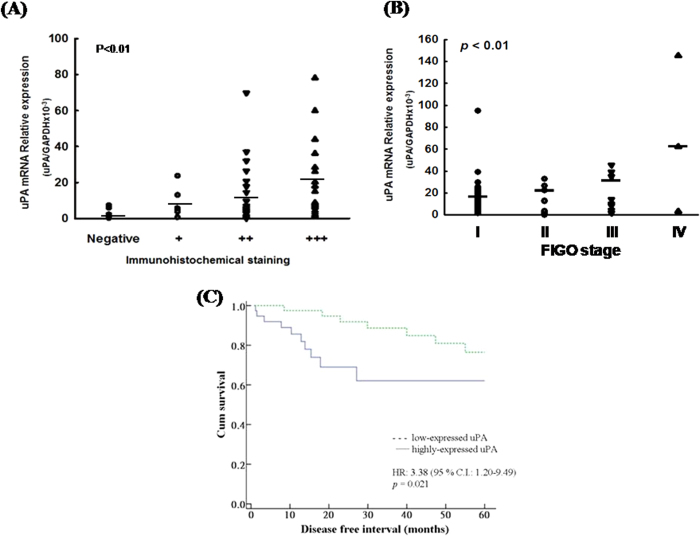
(**A**) The correlation between protein and mRNA expression levels of uPA by immunohistochemistry and RTQ RT-PCR. *Note:* The uPA highly-expressed tumors in IHC staining had significantly higher relative mRNA expressions of uPA (uPA/GAPDH x10^−3^) compared to the uPA low-expressed tumors by IHC staining (strongly-positive tumors: 21.5, moderately-positive tumors: 12.9, weakly-positive tumors: 8.4, and negative tumors: 2.1; *p* < 0.01, by one-way ANOVA). (**B**) The correlation between mRNA expression levels of uPA and tumor stages. *Note:* The tumors of higher FIGO stage had significantly higher relative mRNA expressions of uPA (uPA/GAPDH x10^−3^) than those of lower FIGO stage (17.3 in stage 1, 22.2 in stage 2, 34.9 in stage 3, and 62.0 in stage 4; p < 0.01, by one-way ANOVA). (**C**) Survival curves of disease free survival (DFS) of the 169 endometrial endometrioid adenocarcinoma patients in highly- and low-expressed uPA groups by a cut-off value of uPA/GAPDH of 0.015. *Note:* The patients with highly-expressed uPA had significantly shorter DFS compared with those with low-expressed uPA (HR: 3.38 (1.20-9.49), *p* = 0.021, Kaplan-Meier).

Correlations were found between protein and mRNA expression levels of uPA in immunohistochemistry and RTQ RT-PCR (Fig. 2A). The uPA highly-expressed tumors in IHC staining had significantly higher relative mRNA expressions of uPA (uPA/GAPDH x10^−3^) compared to the uPA low-expressed tumors by IHC staining (strongly-positive tumors: 21.5, moderately-positive tumors: 12.9, weakly-positive tumors: 8.4, and negative tumors: 2.1; *p* < 0.01, by one-way ANOVA).

The correlations between the mRNA levels of uPA and tumor stage were further evaluated. The tumors of higher FIGO stage had significantly higher relative mRNA expressions of uPA (uPA/GAPDH x10^−3^) than those of lower FIGO stage (17.3 in stage 1, 22.2 in stage 2, 34.9 in stage 3, and 62.0 in stage 4; p < 0.01, by one-way ANOVA) (Fig. 2B).

The results indicated that endometrial cancer cells could secrete uPA, and that tumors that expressed higher mRNA levels of uPA could also secrete higher amounts of uPA protein in EEC tissues. And advance-staged tumors could secrete higher amounts of uPA than early-staged tumors.

### Prognostic value of uPA in the 169 EEC patients

We further evaluated the impact of an increased uPA expression in cancerous tissues on disease-free survival (DFS) of the EEC patients. In this study, 17 patients had disease relapse at the time of analysis. By referencing the expression of GAPDH, we further divided the patients into highly-expressed (51 cases, uPA/GADPH > 0.015) and low-expressed (118 cases, uPA/GADPH < 0.015) uPA groups. The patients with highly-expressed uPA had a significantly shorter DFS (HR: 3.38 (1.20-9.49), *p* =  0.021, log-rank test) compared with those with low-expressed uPA. The corresponding survival curves of DFS of the highly- and low-expressed uPA groups are presented in Fig. 2C.

The prognostic factors for DFS of the 169 EEC patients are shown in Table 4. Neither age, tumor grade, nor the depth of myometrial invasion had a significant effect on DFS. However, advanced FIGO stage (stage III/IV versus stage I/II, HR: 3.14 (95% CI: 1.60-7.14), *p* = 0.01), lymph node metastasis (yes versus no, HR: 4.51 (95% CI: 1.21-16.83), *p* = 0.025), and an increased expression level of uPA (high versus low, HR: 3.38 (95% CI: 1.20-9.49), *p* = 0.021) had a significantly poor impact on the DFS in univariate analysis. In multivariate analysis, an increased uPA level (high versus low, HR: 4.65 (95% CI: 1.16-11.5), *p* = 0.030) was the only independent poor prognostic factor for DFS.

### Expression of uPA correlated with the migratory capabilities of endometrial tumor cells

**Figure 3:**
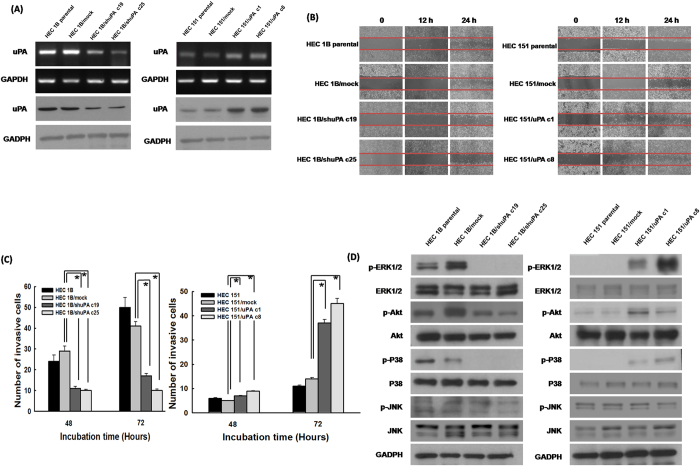
The expression level of uPA correlated with the migration and invasion of endometrial tumor cells and the phosphorylation of ERK1/2, Akt and p38 phosphorylation. (**A**) RT-PCR and Western blot analyses of uPA expression in various endometrial tumor cells and their transfectants. *Note:* The expression levels of uPA decreased in HEC1B/shuPA c19 and HEC1B/shuPA c25 cells compared to those in HEC1B parental and HEC1B/mock cells. However, the expression levels of uPA in HEC151/uPA c1 and HEC151/uPA c8 cells were higher than those in HEC151 parental and HEC151/mock cells. (**B**) Representative images of migration assays in various endometrial tumor cells and their transfectants. *Note:* Both HEC1B/shuPA c19 and HEC1B/shuPA c25 cells with a low uPA expression showed significantly fewer migrating cells than parental HEC1B and HEC1B/mock cells. However, both HEC151/uPA c1 and HEC151/uPA c8 cells with a high uPA expression had more migrating cells than the parental HEC151 and HEC151/mock cells. (**C**) Bar graph depicting the number of invasive cells by Boyden chamber analysis. *Note:* The low uPA-expressing HEC1B/shuPA c19 and HEC1B/shuPA c25 cells had a lower number of invasive cells than the parental HEC1B or HEC1B/mock cells (* indicates p < 0.001, one-way ANOVA). However, the high uPA-expressing HEC151/uPA c1 cells and HEC151/uPA c8 cells had a 2-fold higher number of invasive cells at 72 hours compared with the parental HEC151 and HEC151/mock cells (* indicates, p < 0.001, one-way ANOVA). (**D**) Western blotting analysis of phosphorylation of ERK1/2, Akt, JNK and p38 molecules. The phosphorylation of ERK1/2, Akt and p38 was consistently downregulated when the expression of uPA in tumor cells (HEC1B/shuPA c19 and HEC1B/shuPA c25 cells) decreased. The uPA highly-expressing tumor cells (HEC151/uPA c1 and HEC151/uPA c8 cells) revealed higher ERK1/2, Akt and p38 phosphorylation.

We first evaluated the expression levels of uPA in various endometrial tumor cell lines and transfectants. The expression levels of uPA were decreased in several uPA knockdown HEC1B transfectants such as HEC1B/shuPA c19 and HEC1B/shuPA c25 cells compared to those in HEC1B parental and HEC1B/mock cells by RT-PCR and Western blotting (Fig. 3A, left panel). However, the expression levels of uPA in several uPA over-expressed transfectants such as HEC151/uPA c1 and HEC151/uPA c8 were higher than those in HEC151 parental and HEC151/mock cells by RT-PCR and Western blotting (Fig. 3A, right panel).

We then investigated if the expression of uPA correlates with cell migration of endometrial cancer cells. The representative figures of the cell migration assays are shown in Fig. 3B. Both the HEC1B/shuPA c19 and HEC1B/shuPA c25 cells which had a lower uPA expression showed significantly fewer migrating cells than the parental HEC1B and HEC1B/mock cells (Fig. 3B, left panel). However, both the HEC151/uPA c1 and HEC151/uPA c8 cells which expressed a higher level of uPA had more migrating cells than the parental HEC151 and HEC151/mock cells (Fig. 3B, right panel).

These results suggested that uPA could enhance the migration of endometrial cancer cells.

### uPA highly-expressing endometrial tumor cells had higher invasive capabilities

To investigate whether the expression of uPA also correlates with the invasive capability of endometrial cancer cells, Boyden chamber assays performed. The low uPA-expressing HEC1B/shuPA c19 and HEC1B/shuPA c25 cells had a lower number of invasive cells than the parental HEC1B or HEC1B/mock cells (Fig. 3C, left panel). Furthermore, the high uPA-expressing HEC151/uPA c1 cells or HEC151/uPA c8 cells had a 2-fold higher number of invasive cells at 72 hours compared with the parental HEC151 and HEC151/mock cells (p < 0.001, one-way ANOVA; Fig. 3C right panel).

These results implied that uPA might also enhance the invasive activity of endometrial cancer cells.

### uPA could enhance the ERK1/2, Akt and p38 signaling pathways of endometrial tumor cells

To investigate the possible signaling pathways induced by uPA in endometrial cancer cells, immunoblotting of the phosphorylation of various molecules was performed in endometrial tumor cell lines and their transfectants. The phosphorylation of ERK1/2, Akt and p38 was consistently down-regulated when the expression of uPA in the tumor cells (HEC1B/shuPA c19 and HEC1B/shuPA c25 cells) decreased (Fig. 3D, left panel). In addition, the uPA highly-expressing tumor cells (HEC151/uPA c1 and HEC151/uPA c8) had higher levels of ERK1/2, Akt and p38 phosphorylation (Fig. 3D, right panel). However, the level of phosphorylation of JNK did not change in the uPA over-expressing or down-regulated tumor cells (Fig. 3D).

**Figure 4:**
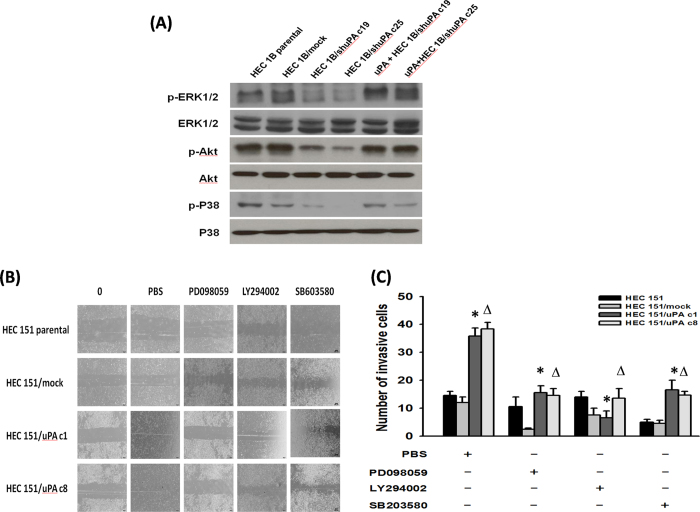
uPA could enhance the ERK1/2, Akt, or p38 phosphorylation and the migration and invasion of endometrial tumor cells could be blocked by respective inhibitor. (**A**) Western blotting analysis of phosphorylation of ERK1/2, Akt, JNK and p38 molecules in HEC1B/shuPA c19 and HEC1B/shuPA c25 cells treated with recombinant uPA. *Note:* The phosphorylation of ERK1/2, Akt and p38 was consistently upregulated in cells treated with recombinant uPA. (**B**) Representative images of migration assays in HEC151 parental cells and their uPA transfectants treated with respective ERK1/2, Akt, or p38 phosphorylation inhibitor. *Note:* Both HEC151/uPAc1 and HEC151/uPAc8 cells had fewer migrating cells when treated with respective inhibitor than those treated with PBS alone. (**C**) Bar graph depicting the number of invasive cells of HEC151 parental cells and their uPA transfectants treated with respective ERK1/2, Akt, or p38 phosphorylation inhibitor by Boyden chamber analysis. *Note:* Lower numbers of invasive cells in HEC151/uPAc1 and HEC151/uPAc8 cells treated with respective inhibitor than those treated with PBS alone. (*, Δ indicate p < 0.001, one-way ANOVA).

The expressions of ERK1/2, Akt and p38 phosphorylation in HEC1B/shuPA c19 and HEC1B/shuPA c25 cells treated with or without recombinant uPA were further evaluated. The expressions of ERK1/2, Akt and p38 phosphorylation in HEC1B/shuPA c19 and HEC1B/shuPA c25 cells were significantly enhanced, when treated with recombinant uPA (Fig. 4A).

Our results revealed that uPA can enhance the ERK1/2, Akt and p38 signaling pathways in endometrial cancer cells.

### Blockade of ERK1/2, Akt or p38 siginal pathways could suppress the migrating and invasion capabilities of uPA highly-expressing endometrial tumor cells

The influences of specific inhibitors on the cell migration and invasion capabilities of HEC 151 uPA-transfectants were further evaluated. As shown in Fig. 4B, the cell migration capabilities of HEC151/uPA c1 and HEC151/uPA c8 cells were significantly reduced regardless treated with PD098059, LY294002, or SB603580. Besides, the numbers of invasive cells of HEC151/uPAc1 and HEC151/uPAc8 cells treated with respective inhibitor were significantly fewer than those treated with PBS alone (Fig. 4C).

Our results indicated that uPA might promote the migration and invasion of endometrial cancer cells through ERK1/2, Akt and p38 signaling pathways.

## Discussion

Genomic and proteomic technologies have significantly accelerated the discovery of potential DNA, RNA, and protein biomarkers involved in cancer development or progression^11^. The present study used a combination of high-throughput RNA assays, multi-step validation with SQ RT-PCR and RTQ RT-PCR, and relevant IHC assays to confirm that uPA was expressed in EEC tissues. Moreover, both the mRNA and protein expression levels of uPA were significantly higher in the EEC patients with deep myometrial invasion and lymph node metastasis than in those without deep myometrial invasion or lymph node metastasis. This indicated that uPA may be an emerging marker for the diagnosis and prognosis of EEC.

In this study, several strategies were used to filter out potential genes that may be involved in tumorigenesis, progression, and metastasis of EEC. First, pooled sample expression profiling was used^12^. cDNA micro-array analysis is an important tool in studying gene expression profiles in genomic research, and a pooling approach helps to improve the efficiency of the profiling using fewer microarray chips and ensuring more robust measurement of transcript levels^13^. The benefits of sample pooling are multiple. By pooling tissue specimens, the RNA requirement per tumor is proportionately reduced, making sample pooling attractive when tissue banks can provide only limited quantities of tumor samples per patient.

In addition, the risk of a single specimen contributing bias to the pool is also proportionately reduced by increasing the sample size. Furthermore, because the number of micro-array chips necessary for the study is reduced, the computational requirements for analysis are also reduced^12^. When 5-10 samples are used to construct a pool, informative data can be mined regarding the identification of both tumor markers and progression markers^12^. Using the advantages of the pooling strategy, 41 candidate tumor progression markers were identified. After validation with SQ RT-PCR, uPA, PLOD2, and five other genes were confirmed to be over-expressed in the EEC tissues.

Second, large training and testing sets that shared the same distribution for validation were used. Separating data into training and testing sets is an important part of evaluating data mining models. Using similar data for training and testing, the effects of data discrepancies are minimized and the characteristics of the model better understood^14^. After validating with the training set, only uPA and PLOD2 were confirmed to be associated with the tumorigenesis of EEC. The correlation of uPA with EEC tumor invasion and metastasis were further established in the testing set.

The uPA gene encodes a serine protease involved in the degradation of the extracellular matrix and possibly tumor cell migration and proliferation by regulating the plasminogen/plasmin system. uPA has also been reported to contribute to anti-thrombolytic activity to remove blood clots, and to help stimulate angiogenesis in tumor cells^15^. uPA expression has been reported to be up-regulated in many types of human cancers, including esophageal cancer^16^, colorectal cancer^17^,^18^, prostate cancer^19^,^20^, and breast cancer^21^,^22^. Its expression level has also been correlated with cancer progression and metastasis. However, relatively little is known about its role in endometrial cancer^23^,^24^,^25^.

This study is the first to demonstrate an association between increasing uPA expression and tumor invasion and metastatic abilities in endometrial cancer. The results of this study provide proof that uPA is more highly-expressed in cancerous tissues than in hyperplastic and normal endometrium, which is consistent with previous studies^23^,^24^,^25^. The current study further demonstrated that uPA is significantly elevated in patients with features of biologically aggressive endometrioid carcinomas. Tumors expressing high levels of uPA had a higher incidence of deep myometrial invasion, LVSI, lymph node metastasis, and advanced tumor stage. In addition, the IHC studies suggested that endometrial tumor cells secrete more uPA protein to promote local invasion and distant metastasis. A higher expression of uPA in endometrial endometrioid adenocarcinoma could independently predict a poor DFS of the cancer patients in this study (Table 5 and Fig. 2C).

The mechanisms of uPA involvement in tumor invasion and metastasis have been investigated in other malignancies, and especially breast cancer. uPA is involved in the regulation of breast cancer invasion and metastasis through its ability to facilitate degradation of the extracellular matrix, cell proliferation, angiogenesis, migration, and adhesion^22^,^26^. However, the mechanism by which uPA influences the tumorigenesis, tumor invasion, and metastasis of EEC is unclear. Our results highlight the importance of uPA in EEC and its significance for further biological investigations. Moreover, our results imply that uPA may be a candidate biomarker marker with clinical utility for the diagnosis, management, and possibly target-based therapy for endometrial cancer in the future.

The PLOD2 gene has also been found to be a good marker for differentiating endometrial cancer from normal endometrium and atypical hyperplastic endometrium. Procollagen-lysine, 2-oxoglutarate 5-dioxygenase (PLOD) genes are involved in fibrotic processes and tissue remodeling, and are also known to be lysyl hydroxylases that catalyze the hydroxylation of lysyl residues as a post-translational event in collagen biosynthesis^27^. Three isoforms of the enzyme have been characterized: PLOD1, PLOD2, and PLOD3^28^. Among the PLOD genes, PLOD2 contributes to tumor angiogenesis and cancer prognosis.

The correlation between PLOD2 and solid tumors has been reported in several studies. Noda *et al.* suggested that PLOD2 expression is a significant, independent factor of a poor prognosis in patients with hepatocellular carcinoma^29^. Dong *et al.* also reported that PLOD2 expression can provide significant prognostic information in patients with glioblastoma^30^. Arao *et al.* investigated the role of ZD6474, an agent that selectively targets vascular endothelial growth factor receptor-2 tyrosine kinase and epidermal growth factor receptor tyrosine kinase in a metastatic gastric cancer model^31^. They found that the PLOD2 gene might be a useful biomarker for monitoring the effects of ZD6474 treatment. However, the association between PLOD2 and EEC has not previously been reported in the literature. Further prospective studies with a sufficient number of patients are warranted to investigate the role of PLOD2 in EEC.

The present study has some limitations. First, the average follow-up was short (median follow-up, 3.3 years). Because of the good prognosis of the patients with endometrial carcinomas who undergo surgery, overall survival could not be evaluated due to the limited number of patients who died. We could only demonstrate that uPA expression is an independent prognostic factor for DFS. Nevertheless, larger studies with more cases of mortality and a longer follow-up period may provide a more definitive statement regarding the association of uPA levels with endometrial cancer survival. Second, plasma uPA levels have been reported to be associated with cancer invasion, progression, and metastasis in prostate cancer, however, they were not checked in this study^20^,^32^. Circulating uPA is an easier and more convenient tool for the diagnosis and treatment of endometrial cancer. Further studies are therefore warranted to investigate the correlation of plasma uPA level with clinicopathological parameters such as FIGO stage, histological grade, depth of myometrial invasion, and lymph node status with endometrioid carcinoma.

uPA activates the ERK, Akt and p38 signaling pathways to execute its biological function. The aggressiveness of invasion and migration of tumor cells determines the metastatic potential that remains a significantly poor prognostic feature of endometrium cancer^33^. The over-expression of uPA and its receptor (uPAR) has been well documented in a wide variety of tumor cells, and it was reported that uPA/uPAR expression is highly correlated with tumor invasion and metastasis in a breast cancer model^34^. However, the role of uPA in the invasion of endometrial cancer remains poorly understood. Our results identified that uPA could up-regulate the phosphorylation of ERK, Akt and p38, resulting in the migration and invasion of endometrial cancer cells (Fig. 3 and 4). ERK and JNK are known as so-called mitogenic pathways in mammalian cells which induce cell migration and invasion^35^. However, the phosphorylation of JNK was not observed in uPA highly-expressing endometrial tumor cells in the present study, and the endometrial cancer cells expressed uPA to enhance migratory and invasive capabilities through the ERK pathway alone.

The Akt pathway is also an important pathway involved in the invasion and migration of endometrial cancer cells. To the best of our knowledge, this study is the first to identify that Akt can be regulated by uPA. Kim *et al.* reported that Akt/PKB up-regulates metalloproteinase-9 to enhance the invasive abilities of cancer cells^36^. However, whether uPA also regulates the Akt-mediated signaling cascade remains unclear. Further studies are required to address the specific events that occur upstream and downstream of Akt related to the invasion of endometrial cancer.

Targeted therapy can be an effective cancer therapeutic strategy via the ERK or Akt signaling pathways. These two pathways are activated by a wide variety of mitogenic stimuli that interact with distinct receptors and turn on biological behaviors, including mitosis^37^, differentiation^38^, and tumor progression^39^. Our results provide potential targets to specifically block the ERK, Akt or p38 pathway to treat endometrial cancer. With the universal role of ERK, Akt and p38 pathways in regulating normal cellular functions, sensitive and specific inhibitors for these pathways could be tested in future clinical trials.

In conclusion, our study provides new evidence that uPA is involved in the tumorigenesis of EEC, and that a higher uPA expression is associated with tumor invasion and metastasis, and poorer DFS. uPA can enhance the migratory and invasive capabilities of endometrial cancer cells through the ERK, Akt or p38 pathway. Targeting these signaling pathways may be a potential strategy for the therapy of endometrial cancer.

## Methods

### Patients and samples

The methods were carried out in accordance with the approved guidelines. The Institutional Review Board of the two hospitals (NTUH, CGH) approved the experimental protocols, and all patients provided informed consent before surgery. From March 2007 to December 2010, 169 women with EEC who received staging surgery were enrolled from the National Taiwan University Hospital and Cathay General Hospital. Normal proliferative endometrial tissues (NEM) from 20 women with benign diseases (i.e., uterine myoma and adenomyosis) were obtained as the normal controls. Another 20 women with atypical endometrial hyperplasia (AEH) who underwent a hysterectomy were also included. The inclusion criteria for all cases were: (i) unambiguous histology and absence of mixed tumor type; (ii) sufficient tissue available for RNA extraction; and (iii) absence of any treatment prior to surgery.

**Figure 5:**
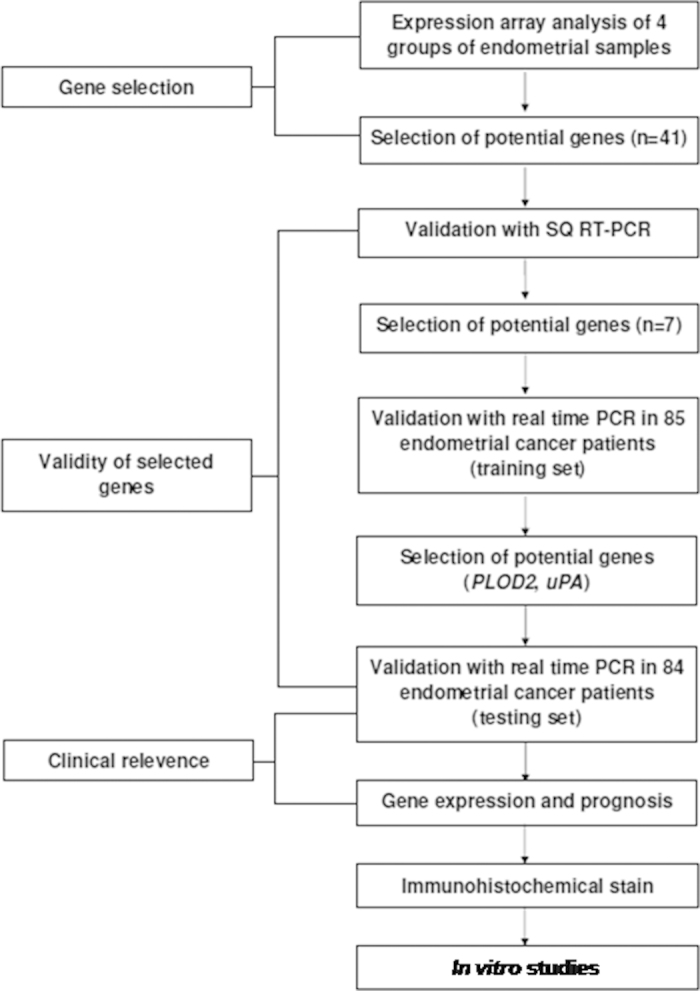
The flow chart of the study. Forty-one candidate genes were first selected by expression assay analysis, and then 7 genes were validated by SQ RT-PCR. The mRNA levels of these 7 gens were first studied in the training set (n = 85) by RTQ RT-PCR. Two potential genes (PLOD2 and uPA) selected from the training set were further investigated in the testing set (n = 84). The relevance between the clinicopathological parameters and outcomes of the patients were correlated with the mRNA expression of the genes. The protein and mRNA levels of uPA in the endometrial cancerous tissues were then correlated by immunohistochemical staining and RTQ RT-PCR. Finally *in vitro* experiments related to uPA in endometrial carcinogenesis were performed. SQ RT-PCR, semi-quantitative reverse transcriptase polymerase chain reaction; RTQ RT-PCR, real-time quantitative reverse transcriptase polymerase chain reaction.

Tissue specimens collected during the hysterectomies were immediately frozen in liquid nitrogen and stored at −80 °C until analysis. The staging, histology, and grading criteria of endometrial cancer were based on the 2009 International Federation of Obstetrics and Gynecologic (FIGO) surgical staging system^40^. Histology was reviewed by a gynecological pathologist (K.T. Kuo). Pre-existing clinical data including age, body mass index, menopausal status, recurrence status, adjuvant therapy, and survival, were collected and entered into a database. Progression-free survival was measured as the period from surgery to the date of confirmed recurrence or disease progression, or to the date of the investigators’ last record of a disease-free status. The detailed medical records were examined until December 31, 2012. The study flow chart is shown in Fig. 5.

### Extraction of RNA from the tissues of NEM, AEH, and EEC

The total RNA from NEM, AEH, and EEC tissues was isolated using TRIzol reagent (Invitrogen, Carlsbad, CA) following the manufacturer’s instructions. The integrity of the RNA was evaluated using 18S and 28S ratios and RNA integrity numbers using a Nanodrop ND-1000 spectrophotometer (Nanodrop Technologies, Wilmington, DE).

### Gene expression analysis

Gene expressions in the NEM, AEH, and EEC tissues were analyzed by micro-array. Samples were grouped according to stage but selected randomly from the list of banked, frozen tissues. Ten NEM, 10 AEH, and 20 EEC specimens of early and advanced stages were used for the micro-array experiments (Group 1, early-stage EEC [stages I and II] (n = 10) and Group 2, advanced-stage EEC [stages III and IV] (n = 10). Samples were pooled in equimolar amounts (10 samples per pool) for micro-array analysis.

Isolated RNA was first reverse-transcribed and the resulting complementary DNA was used for *in vitro* transcriptional synthesis of fluorescent-labeled nucleic acid probes according to the manufacturer’s instructions (Affymetrix Inc., Santa Clara, CA). Micro-array analysis was performed using an Affymetrix GeneChip Human Genome U133 Plus 2.0 Array (Affymetrix) that consisted of over 54,000 transcripts covering more than 38,500 well-characterized genes. Eleven pairs of oligonucleotide probes were used to measure the level of transcription of each sequence. The entire process was done according to the manufacturer’s (Affymetrix) instructions.

Briefly, double-stranded cDNA was synthesized by a chimeric oligo-nucleotide with oligo-dT and T7 RNA polymerase. Amplification and labeling were monitored using a GeneChip Eukaryotic Poly-A RNA Control Kit (Affymetrix) with exogenous positive controls that were spiked into the total RNA before cDNA synthesis. Samples were hybridized onto a Human Genome U133 Plus 2.0 Array at 45 °C at 60 rpm for 16 hours in a Hybridization Oven 640 (Affymetrix).

Micro-array scanned images were obtained at high-resolution using GeneChip Operating Software and a GeneChip Scanner 3000 (Affymetrix) using the default settings. Raw intensity values were exported for data processing and analysis in Team and Bioconductor^41^,^42^. Data quality and sample relationships were assessed using the Bioconductor Affy, affyQCReport, GCRMA, and pre-process Core packages. Micro-array quality control was performed using affyQCReport. All four samples passed the quality control criteria. GCRMA was used for quantile normalization of different chips in order to compare samples with minimum non-biological differences^43^. As recommended by Shi *et al.*, differentially expressed genes were chosen based on a high-fold change (at least four-fold change in at least one group comparison)^44^. In total, 41 candidate genes were selected.

### Semi-quantitative RT-PCR

To validate the gene expression levels in micro-array analysis, semi-quantitative RT-PCR (SQ RT-PCR) was performed for the first step of validation. Four NEM, 4 AEH, 4 early-staged EEC, and 4 advanced-staged EEC specimens were randomly selected from the banked, frozen tissues. SQ RT-PCR was performed as previously described, with some modifications^45^. Briefly, mRNA was reverse transcribed to cDNA using a Moloney murine leukemia virus reverse transcriptase kit (Invitrogen Life Technologies, San Diego, CA). Glyceraldehyde-3-phosphate dehydrogenase (GAPDH) was used as the housekeeping gene to compare with the 41 candidate genes based on the micro-array results. The PCR products were then analyzed in 1% agarose gel with ethidium bromide staining in TBE solution. The gel images were obtained using a CCD camera (Biocapt Company, Vilbert Lourmat, Marne la Vallee, France), and the bands of interest were stored as TIFF files using BioCapt software as described previously^46^.

The density of the target genes/the density of GAPDH was regarded as the expression level of the target genes in each endometrial tissue sample. Seven potential genes regarded as being tumorigenesis-related were then selected for further analysis.

The urokinase-type plasminogen activator (uPA) gene expression in various cell lines was detected by RT-PCR in different experiments. Briefly, 1 μl of the reaction mixture was amplified by PCR using the following primers: human *uPA*, 5’-GCCATCCCGGACTATACAGA-3’ (sense) and 5’- AGGCCATTCTCTTCCTTGGT-3’ (antisense); *GAPDH* (glyceraldehyde-3-phosphate dehydrogenase), 5’-ACCCAGAAGACTGTGGATGG-3’ (sense) and 5’-TGCTGTAGCCAAATTCGTTG3’ (antisense). Briefly, total RNA of various cell lines was isolated using a TRIzol^®^ RNA isolation kit following the manufacturer’s instructions (Invitrogen). Reverse-transcribed cDNA products were amplified by PCR with specific primers as described above. The amplification products were separated by 1% agarose gel electrophoresis and visualized after staining with ethidium bromide.

### Real-time quantitative RT-PCR

The 169 EEC specimens and the 20 NEM and 20 AEH specimens were randomly assigned to either the training set or the testing set (Table 1). The mRNA expression levels of seven genes, including COL1A2, uPA, PSAT1, SLC2A1, GCLM, TTYH2, and PLOD2 were further examined by TaqMan real-time quantitative (RTQ) RT-PCR in 10 NEM, 10 AEH, and 85 EEC specimens in the training set.

RTQ RT-PCR was performed as described previously^45^,^47^. Briefly, 2 μl of the cDNA 1:10 diluted and TaqMan real-time primers and probes were used for amplification (Applied Biosystems, Foster City, CA). Assay IDs for the genes were as follows: COL1A2, Hs00164099_m1; uPA, Hs01547054_m1; PSAT1, Hs01107691_g1; SLC2A1, Hs00892681_m1; GCLM, Hs00157694_m1; TTYH2, Hs00560544_m1; and PLOD2, Hs01118190_m1. All PCRs were performed in TaqMan Universal PCR master mix (ABgene, Rochester, NY) and an ABI Prism 7900-HT Sequence Detection System (Applied Biosystems). The relative mRNA expression levels of individual genes for each sample were normalized to compare RNA against GAPDH expressions using the comparative Ct (2^−ΔΔCt^) method as described previously^45^.

### Immunohistochemical staining

For the immunohistochemical (IHC) staining of uPA protein expression, 86 specimens were randomly selected from one-half of the patients in the training and testing sets (both n = 43). Briefly, formalin-fixed, paraffin-embedded 5-mm thick sections were deparaffinized, re-hydrated, and subjected to antigen retrieval as described previously (Trilogy, Cell Marque, Hot Springs, AR; autoclaved, 10 min)^48^. The source of the primary antibodies was uPA (antibodies-online.com; ABIN191518). Staining was performed using a PromARK Micro-polymer detection kit (Biocare Medical; GHP516 G).

The uPA immuno-expression of the tumors was scored semi-quantitatively according to the percentage of positive tumor cells in cytoplasm staining. The staining intensity was scored based on signal intensity as follows: negative (complete absence of expression), weakly-positive (<33% positive tumor cells), moderately-positive (>33% and <66% positive tumor cells), and strongly-positive (>66% positive tumor cells). Tumors with negative or weakly-positive staining for uPA were defined as low uPA-expressing tumors, while those with moderately or strongly-positive staining for uPA were defined as high uPA-expressing tumors. All measurements were performed by a single pathologist (K.T. Kuo) without knowledge of any clinical or pathological data or RTQ RT-PCR results before interpretation.

### Cell culture and transfection

Human endometrial cancer cell lines including HEC1B and HEC151 were obtained from the JCRB Cell Bank (Japan) in 2013. Various uPA ectopic or knock-down transfectants were generated as described below. The HEC1B and HEC15 cell lines were tested for human identification by STR marker to confirm the cell line authentication. HEC1B and HEC151 cells were confirmed with JCRB Cell Bank DNA Profile (STR), and D5S818(11,13), D7S820(9,11), D16S539(11,12), VWA(18), TH01(6,7), Amelogenin(X), TPOX(8,11), and CSF1PO(10,12) markers for HEC1B, and D7S820 (8,11), D16S539 (9,11), VWA (14,19), TH01 (6,9), Amelogenin (X), TPOX (8,11), and CSF1PO (7,11) for HEC151 cells with a perfect match. The cells were last tested in April 2014.

To generate pcDNA3-uPA, uPA was first amplified with PCR using placenta cDNA as the template, and 5’-CCGGTCTAGAATGAGAGCCCTGCTGGCGCG-3’ and 5’-CGCGGATCCTCAGAGGGCCAGGCCATTC -3’ as the primers. The amplified product was then cloned into the XbaI/BamH1 sites of pcDNA3 vector (Invitrogen Life Technologies). The transfection of uPA was performed using Lipofectamine^TM^ 2000 reagent (Invitrogen Life Technologies) according to the manufacturer’s instructions. To select stable clones, hygromycin (700 μg/ml) was added to the culture medium 48 hours after transfection. The hygromycin-resistant clones were individually picked, expanded and assayed for the expression of the transfected uPA by RT-PCR and flow cytometric analysis. The original and uPA-transfected HEC151 cells were used in the subsequent experiments.

To generate *in-vitro* knockdown uPA, HEC1B/shuPA c19 and HEC1B/shuPA c25 cells were generated by transducing HEC1B cells with the lentiviral vector. Briefly, uPA cDNA was amplified by PCR from human placenta cDNA and cloned into the pLKO/AS3.1.EGFP3 lentiviral vector (Academia Sinica, Taiwan) to generate pLKO/uPA/AS3.1.EGFP3, which, together with pCMVΔR8.91 (Academia Sinica, Taiwan) and pMDG (Academia Sinica, Taiwan) were then transfected into 293T cells to assemble the lentivirus. The lentivirus was collected 48 hours after transfection. The HEC1B cells were further infected by a lentivirus with 8 μg/ml polybrene (Sigma Chemicals Co., St Louis, MO) for 48 hours. A single clone was isolated and cultured for further studies.

### Immunoblotting

Immunoblotting was used to assess the protein expression of uPA in different experiments. Cells were lysed with protein lysis buffer (137 mM NaCl, 2.7 mM KCl, 1 mM MgCl2, 1 mM CaCl2, 1% Nonidet P40, 10% glycerol, 1 mg/ml BSA, 20 mM Tris/HCl (pH 8.0) and 2 mM orthovanadate). The protein extracts were quantified using a BCA (bicinchoninic acid) protein assay kit (Pierce). Then 50 μg of each cell lysate was resolved by SDS/PAGE (12% gel), transferred to a PVDF/nylon membrane (Millipore), and probed with antibodies specific to uPA, β-actin, extracellular signal-regulated kinase (ERK), phospho-ERK, Akt, c-Jun N-terminal kinase (JNK), phospho-JNK, p38, phospho-p38 (Upstate Biotechnology) or phospho-Akt (Ser473, Chemicon International). The membrane was then probed with either horseradish peroxidase-conjugated goat anti-mouse or goat anti-rabbit antibodies. The specific bands were visualized by an enhanced chemiluminescence Western blotting system (GE Healthcare).

The detection of ERK, AKT and p-P38 phosphorylation in the HEC1B/shuPA c19 and HEC1B/shuPA c25 cells treated with uPA were performed. Briefly, the cells were seeded and serum-free medium was replaced overnight, followed by treatment with 20 nM of recombinant uPA (R&D Systems, Minneapolis, MN) for 1 h^49^. The cells were lysed and immunoblotting analysis was performed as described earlier.

### Cell migration assays

Cell migration was determined using wound-healing assays with some modifications^12^. Various cell lines and their transfectants were seeded into six-well plates overnight. A sterile 200 μl pipette tip was used to scratch the plate to generate a wound. The cells were then cultured for 48 hours. Cells which migrated into the wound were defined as migrating cells and were visualized with an inverted Olympus phase-contrast microscope. Different areas in each assay were observed to count the number of migrating cells into the origin of the wound.

The cell migration assays of HEC 151 and their uPA-transfectants treated with 25 μm of respective inhibitor such as PD098059 (ERK inhibitor), LY294002 (PI3K inhibitor), or SB603580 (P38 inhibitor) were further evaluated as described earlier.

### Cell invasion assays

Cell invasion assays were performed by using Boyden chambers with filter inserts (pore size, 8 μm) coated with Matrigel^TM^ (40 μg; Collaborative Biomedical, Becton Dickinson) in 24-well dishes (Nucleopore). Briefly, 2 × 10^5^ cells of various cell lines and their transfectants were seeded in the upper chamber, and the same medium was placed in the lower chamber. The plates were incubated for 24 hours, and the cells were then fixed in methanol and stained with 0.05% Crystal Violet in PBS for 1 hour at room temperature. Cells on the upper side of the filters were removed by cotton-tipped swabs, and the filters were washed with PBS. The cells on the lower side of the filters were defined as invasive cells and counted at ×200 magnification in 10 different fields of each filter.

The cell invasion assays of HEC151 and their uPA-transfectants treated with 25 μm of respective inhibitor such as PD098059 (ERK inhibitor), LY294002 (PI3K inhibitor), or SB603580 (P38 inhibitor) were further evaluated as described earlier.

### Statistical analysis and clinical correlation

Statistical analyses were conducted using Statistical Package of Social Studies (SPSS) software version 13.0 (SPSS Inc., Chicago, IL, USA) for Windows. Comparisons between unpaired groups were made using the Mann-Whitney U test, one-way analysis of variance (ANOVA), chi-square test, or Kruskal-Wallis test, as appropriate. Survival curves were generated using the Kaplan-Meier method, and differences in the survival curves were calculated using the log rank test. Cox’s univariate and multivariate regression analyses were used to evaluate the prognostic factors for progression-free survival. A *p*-value less than 0.05 was considered to be statistically significant.

## Additional Information

**How to cite this article**: Huang, C.-Y. *et al.* Urokinase-type Plasminogen Activator Resulting from Endometrial Carcinogenesis Enhances Tumor Invasion and Correlates with Poor Outcome of Endometrial Carcinoma Patients. *Sci. Rep.* **5**, 10680; doi: 10.1038/srep10680 (2015).

## Supplementary Material

Supporting Information

## Figures and Tables

**Table 1:** Clinico-pathologic features of the training set (n = 85) and the testing set (n = 84).

**Characteristic**	**All patients (n** = **169) No. (%)**	**Training set (n** = **85) No. (%)**	**Testing set (n** = **84) No. (%)**	***p*** **value**
Age (Mean±SD)	54.7 ± 12.2	53.1 ± 13.0	56.4 ± 11.2	0.08*

FIGO stage (2009)				
I	118 (69.8%)	62 (72.9%)	56 (66.7%)	0.32**
IA	93 (55.0%)	50 (58.8%)	43 (51.2%)	
IB	25 (14.8%)	12 (14.1%)	13 (14.5%)	
II	15 (8.9%)	8 (9.4%)	7 (8.3%)	
III	32 (18.9%)	12 (14.1%)	20 (23.8%)	
IV	4 (2.4%)	3 (3.5%)	1 (1.2%)	
Tumor grading				0.13^+^
Grade 1	112 (66.3%)	58 (68.2%)	54 (64.3%)	
Grade 2	24 (14.2%)	15 (17.7%)	9 (10.7%)	
Grade 3	33 (19.5%)	12 (14.1%)	21 (25%)	

Depth of myometrial invasion				
<1/2	110 (65.1%)	58 (68.2%)	52 (61.9%)	0.39^+^
>1/2	59 (34.9%)	27 (31.2%)	32 (38.1%)	

Lympho-vascular space invasion				
Negative	94 (55.6%)	44 (51.8%)	50 (59.5%)	0.62^+^
Positive	67 (39.6%)	34 (40%)	33 (39.3%)	
NA	8 (4.7%)	7 (8.2%)	1 (1.2%)	

Lymph node metastases				
No	135 (79.9%)	72 (84.7%)	63 (75%)	0.12^+^
Yes	34 (20.1%)	13 (15.3%)	21 (25%)	

NA: not available.

^*^by t-test.

^**^by Fisher’s exact test; ^+^by Chi-square test.

**Table 2:** Differentially expressed genes showing increased expression in endometrial endometrioid carcinoma (n = 41).

**Gene Symbol**	**AEH vs. NEM**	**EaEC vs. NEM**	**AdEC vs. NEM**	**AdEC vs. EaEC**
APOL1	0.00	0.34	2.45	2.12
BNIP3	−0.62	4.15	2.11	−2.04
CBS	−0.63	4.23	2.70	−1.52
CCNE2	2.41	4.27	3.13	−1.14
CDKN1A	0.48	1.44	2.52	1.09
CMPK2	1.03	3.98	1.76	−2.22
COL1A2	1.90	−8.05	1.25	9.30
CXCL2	0.57	3.85	2.53	−1.33
E2F3	2.41	2.48	1.95	−0.52
EZH2	2.92	2.97	3.01	0.04
EZR	1.46	3.34	2.01	−1.34
FREM2	0.30	−4.46	4.01	8.47
GCLM	−0.01	2.32	2.12	−0.20
GPT2	0.49	1.40	2.44	1.04
HOMER2	1.13	0.07	2.21	2.14
HOXA9	−0.58	−4.02	2.53	6.55
KIF1A	0.13	2.11	2.19	0.08
LIF	2.02	2.47	2.02	−0.46
LIX1	−1.04	0.13	2.21	2.08
MT1M	−0.28	2.10	1.85	−0.25
NANOS1	−0.09	3.11	2.21	−0.89
OPRK1	−0.98	−1.03	5.66	6.69
PACSIN1	1.88	2.26	2.19	−0.07
PGK1	−0.20	2.51	1.84	−0.67
PLAU (uPA)	0.42	0.90	2.40	1.50
PLCXD2	−0.20	2.66	2.12	−0.54
PLOD2	0.69	3.07	2.13	−0.94
PSAT1	0.44	1.84	3.65	1.81
PTGS2	0.29	0.66	3.50	2.84
PTPRJ	0.15	2.33	1.85	−0.48
RAB11FIP4	−0.11	2.11	2.10	−0.01
RCC1	2.29	1.55	1.75	0.20
RIMKLA	−0.37	2.07	1.77	−0.30
SLC2A1	1.50	4.42	3.09	−1.33
SLC7A1	1.08	0.72	2.17	1.45
ST14	1.40	3.12	2.55	−0.57
STAT1	0.21	1.91	2.26	0.35
TFAP2A	1.44	2.62	4.23	1.61
TTYH2	1.15	2.10	1.88	−0.21
VCAM1	−1.77	−3.94	2.44	6.38
VDR	1.34	1.77	3.16	1.39

Data presented as a log_2_ (fold change)

Abbreviations: NEM, normal endometrium; AEH, atypical endometrial hyperplasia; EaEC, early stage endometrioid carcinoma; AdEC, advanced stage endometrioid carcinoma

**Table 3:** Correlation of mean expression levels (target gene/ GAPDH ^*^ 10^−3^) of 7 genes with clinico-pathologic parameters in the training set.

***Gene Name***	***Gene expression levels***										
	*Clinical conditions*				*Myometrial invasion*				*LVSI*		
	NEM	AEH	EEC	P*	<1/2	≧1/2	*P*+	no		Yes	P+
	N = 10	N = 10									
uPA	2.82	9.04	15.01	<**0.01**	12.9	19.4	**0.028**	13.0		17.2	**0.042**
PLOD2	1.50	2.70	4.23	**0.024**	3.79	5.14	**0.041**	3.66		4.95	0.22
COL1A2	2.12	13.5	6.54	0.34	6.70	6.17	0.82	8.33		4.74	0.14
PSAT1	6.64	2.01	5.20	0.85	6.43	2.59	0.26	7.25		2.50	0.17
SLC2A1	0.19	2.47	3.18	0.52	3.14	3.25	0.92	2.30		4.35	0.06
GCLM	3.70	2.94	3.71	0.94	3.58	4.01	0.69	4.01		3.20	0.45
TTYH2	0.24	0.49	0.94	0.63	1.01	0.84	0.57	1.11		0.79	0.47
	*Tumor grading*				*LN statuus*			*FIGO stage*			
	G1	G2	G3	P*	negative	positive	*P*+	I/II		III/IV	P+
	N = 58	N = 15	N = 12		N = 72	N = 13		N = 70		N = 15	
uPA	15.0	15.1	14.5	0.99	12.1	34.1	<**0.01**	12.7		25.7	**0.017**
PLOD2	3.46	5.28	6.25	**0.039**	4.15	4.75	0.68	4.26		4.12	0.92
COL1A2	7.52	4.67	4.02	0.43	6.35	7.80	0.67	6.56		6.43	0.97
PSAT1	4.74	4.04	8.60	0.66	5.54	2.97	0.58	5.69		3.04	0.52
SLC2A1	2.84	3.51	4.49	0.54	3.02	4.21	0.43	3.07		3.72	0.64
GCLM	3.22	6.20	3.06	0.06	3.72	3.67	0.97	3.73		3.67	0.96
TTYH2	1.15	0.49	0.64	0.35	0.95	0.93	0.97	0.98		0.84	0.78

^*^by Kruskal-Wallis test.

^+^by Mann-Whitney U test.

**Table 4:** Correlation of mean expression levels (target gene / GAPDH ^*^ 10^−3^) of uPA and PLOD2 with clinico-pathologic parameters in the testing set.

***Gene Name***	***Gene expression levels***									
	*Clinical conditions*				*Myometrial invasion*			*LVSI*		
	NEM	AEH	EEC	P*	≤1/2	>1/2	*P*+	no	Yes	P+
	n = 10	n = 10	n = 84		n = 52	n = 32		n = 50	n = 33	
uPA	2.68	8.67	13.72	**<0.01**	10.1	19.4	**0.025**	10.9	18.0	**0.038**
PLOD2	1.73	2.58	4.69	**0.012**	3.99	5.80	0.27	3.98	5.95	0.24
	*Tumor grading*				*LN status*			*FIGO stage*		
	G1	G2	G3	P*	negative	positive	P+	I/II	III/IV	P+
	n = 54	n = 9	n = 21		n = 63	n = 21		n = 63	n = 21	
uPA	12.2	13.0	19.8	0.24	11.5	20.9	**0.018**	11.5	20.9	**0.018**
PLOD2	4.02	7.61	5.20	0.42	4.40	5.80	0.47	4.40	5.80	0.47

^*^by Kruskal-Wallis test.

^+^by Mann-Whitney U test.

**Table 5:** Prognostic factors for disease free interval of the 169 EEC patients.

**Variables**	Univariate analysis HR (95% CI)*	***p*** **value**	Multivariate analysis HR (95% CI)*	***p*** **value**
Age at diagnosis (years, continuous)	1.04 (0.98-1.10)	0.18		
FIGO stage (III & IV vs. I & II)	3.14 (1.60-7.14)	**0.01**	2.68 (0.33-22.1)	0.359
Tumor grade (3 vs. 1&2)	2.65 (0.59-11.9)	0.20		
Myometrial invasion (>1/2 vs. < 1/2)	1.42 (0.48-4.25)	0.53		
Lymph node metastases (yes vs. no)	4.51 (1.21-16.83)	**0.025**	2.33 (0.39-14.0)	0.356
uPA expression level (high vs. low)+	3.38 (1.20-9.49)	**0.021**	4.65 (1.16-11.5)	**0.030**

^*^Cox regression model.

^+^high expressed: uPA/GADPH>0.015, low expressed: uPA/GADPH<0.015.
