# Supplementary material for: Urokinase-type Plasminogen Activator Resulting from Endometrial Carcinogenesis Enhances Tumor Invasion and Correlates with Poor Outcome of Endometrial Carcinoma Patients
Source: Sci Rep. 2015 Jun 2;5:10680. doi: 10.1038/srep10680 (PMC4451531; doi:10.1038/srep10680)

**Urokinase-type Plasminogen Activator Resulting from Endometrial Carcinogenesis Enhances Tumor Invasion and Correlates with Poor Outcome of Endometrial Carcinoma Patients**

Chia-Yen Huang, Ming-Cheng Chang, Wei-Yun Huang, Ching-Ting Huang, Yu-Chien Tang, Hsien-Da Huang, Kuan-Ting Kuo, Chi-An Chen and Wen-Fang Cheng

uPA

E3

E4

A1

A2

A3

A4


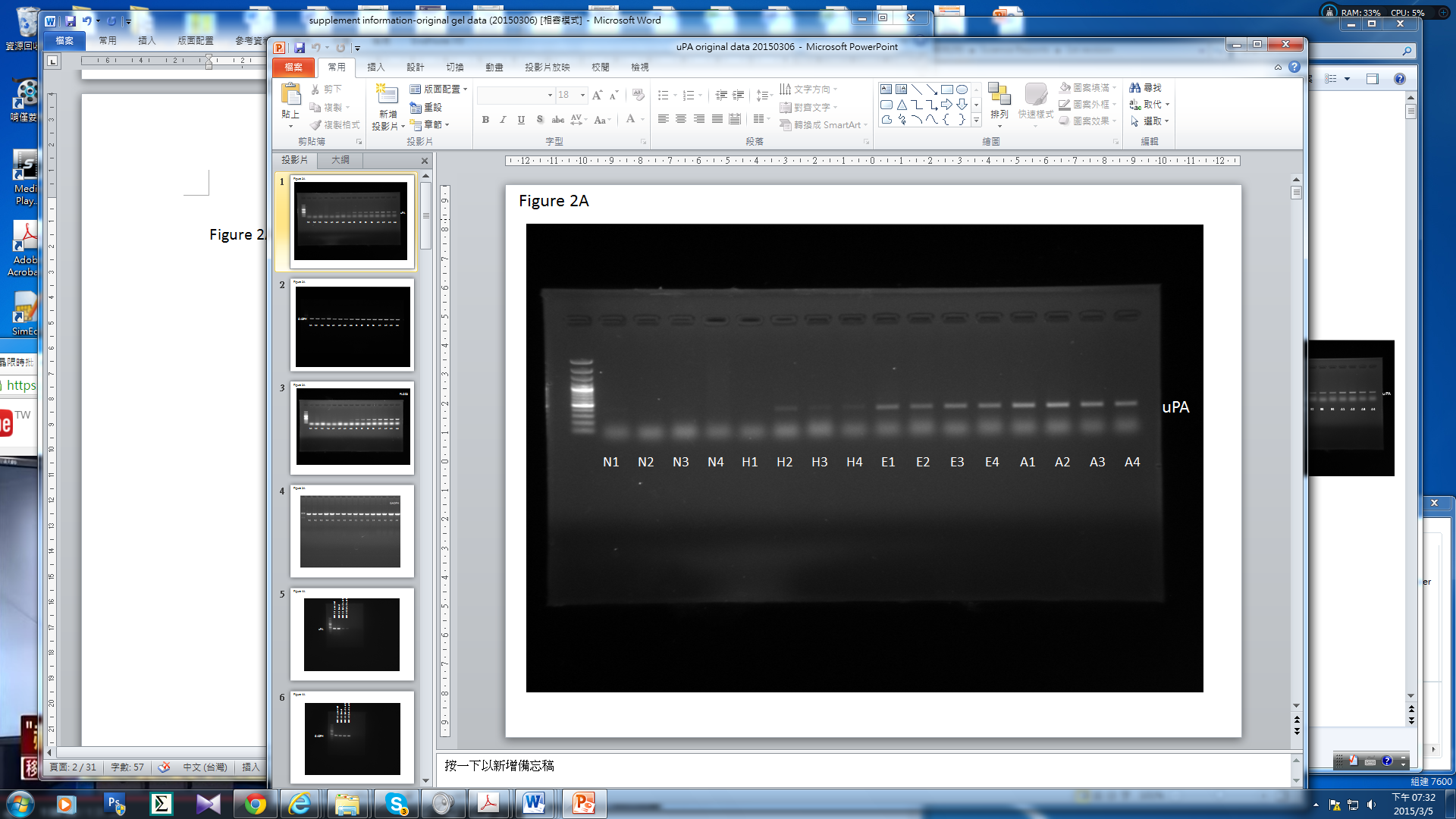


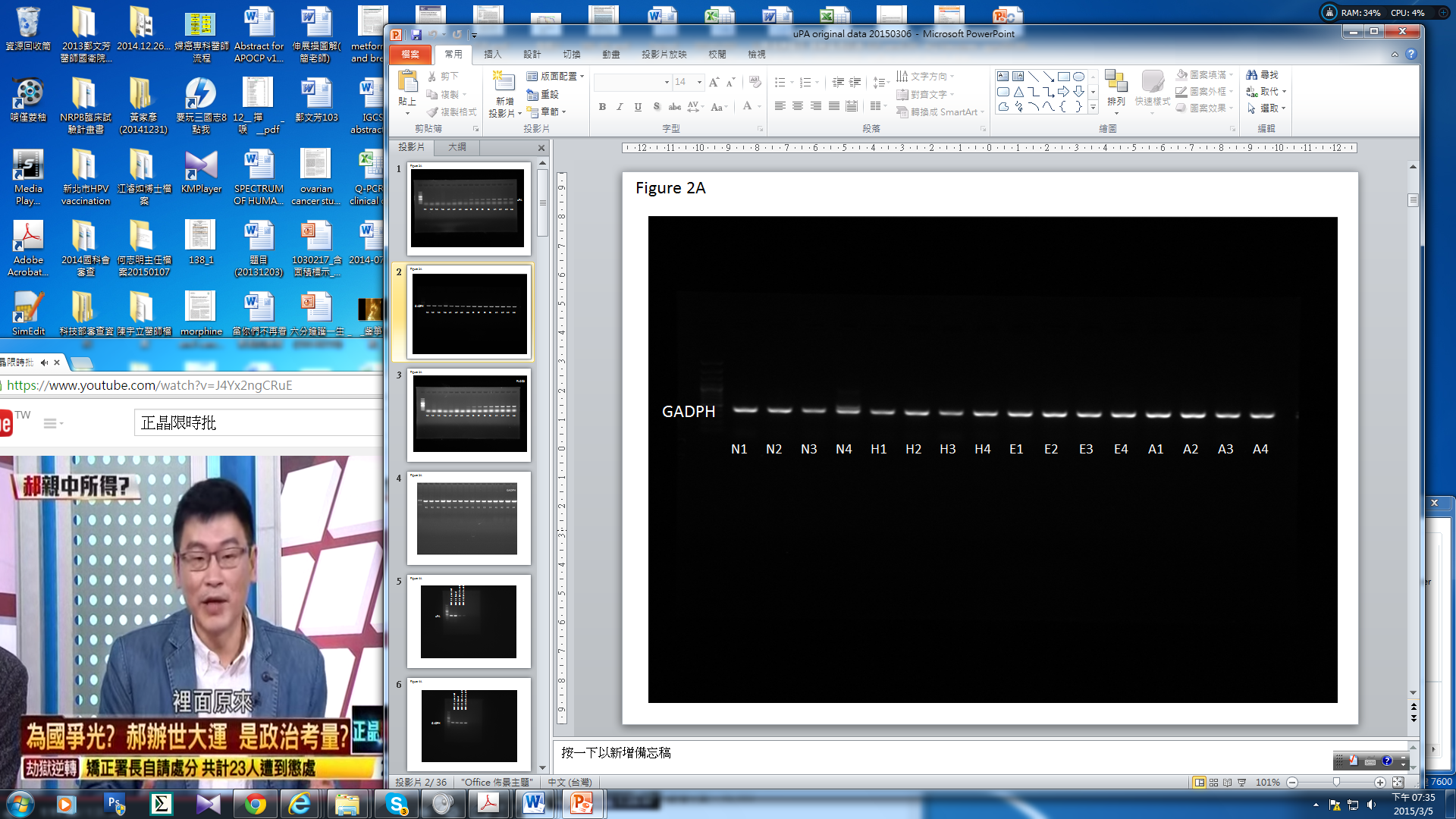


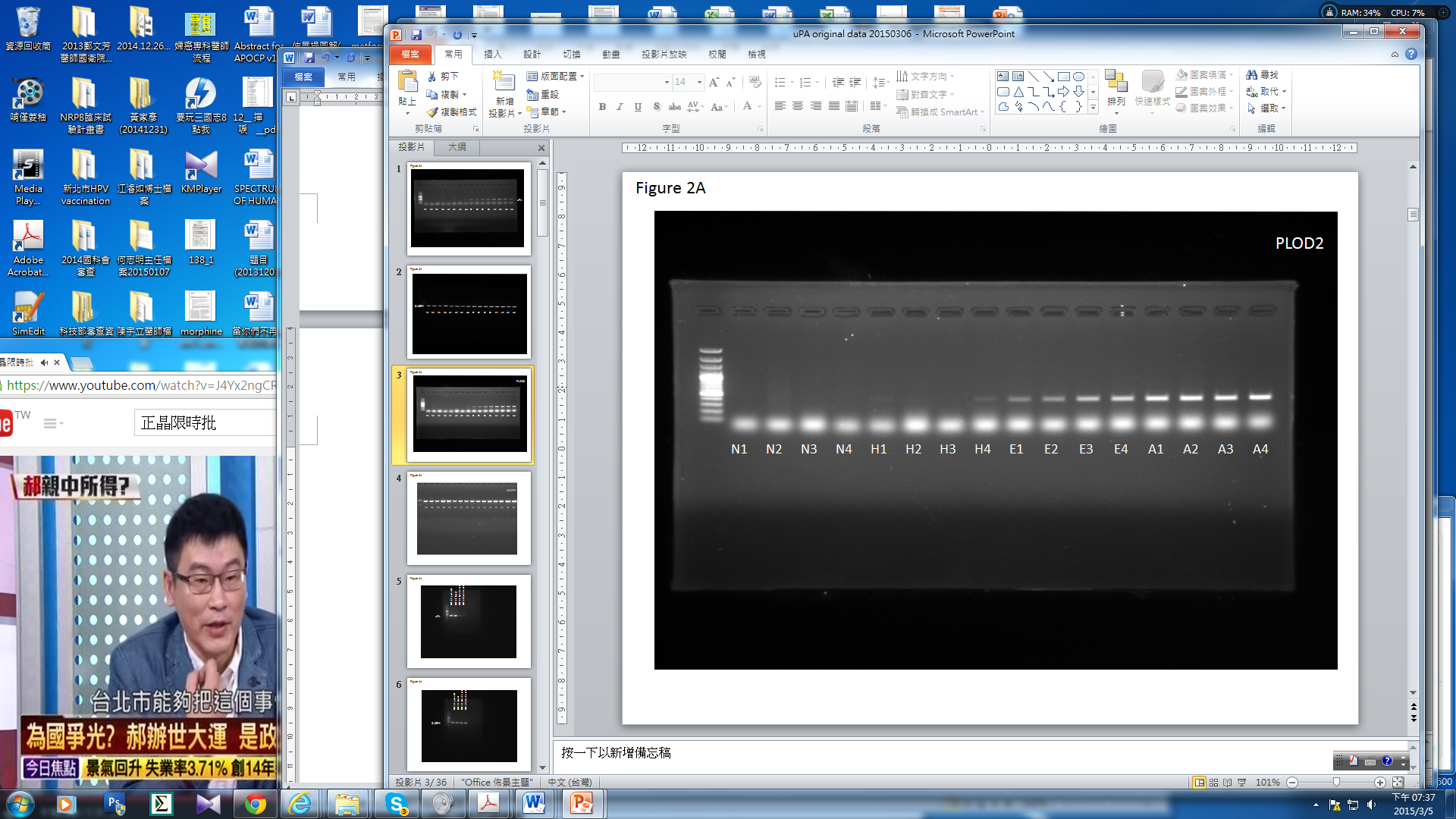


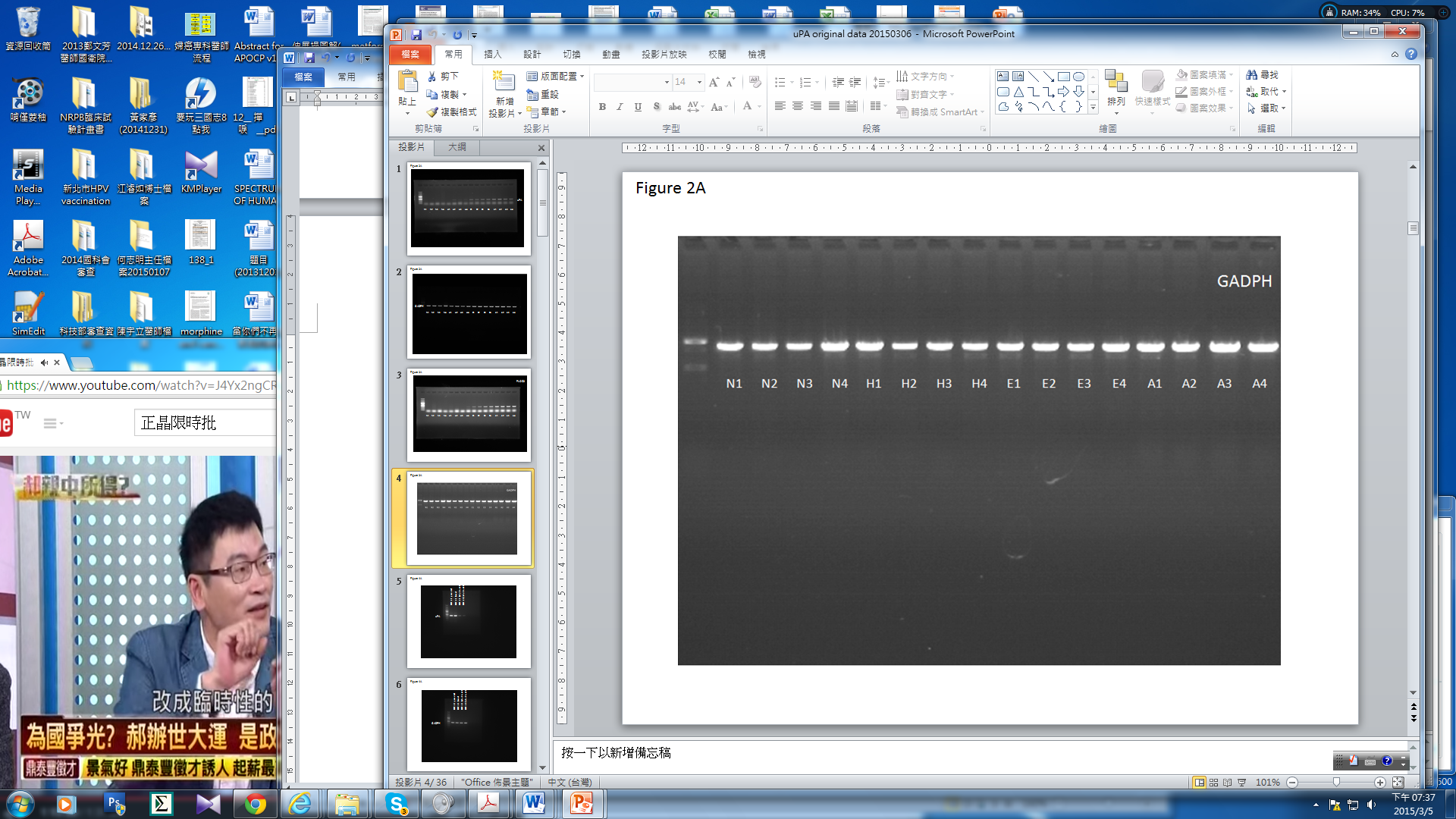


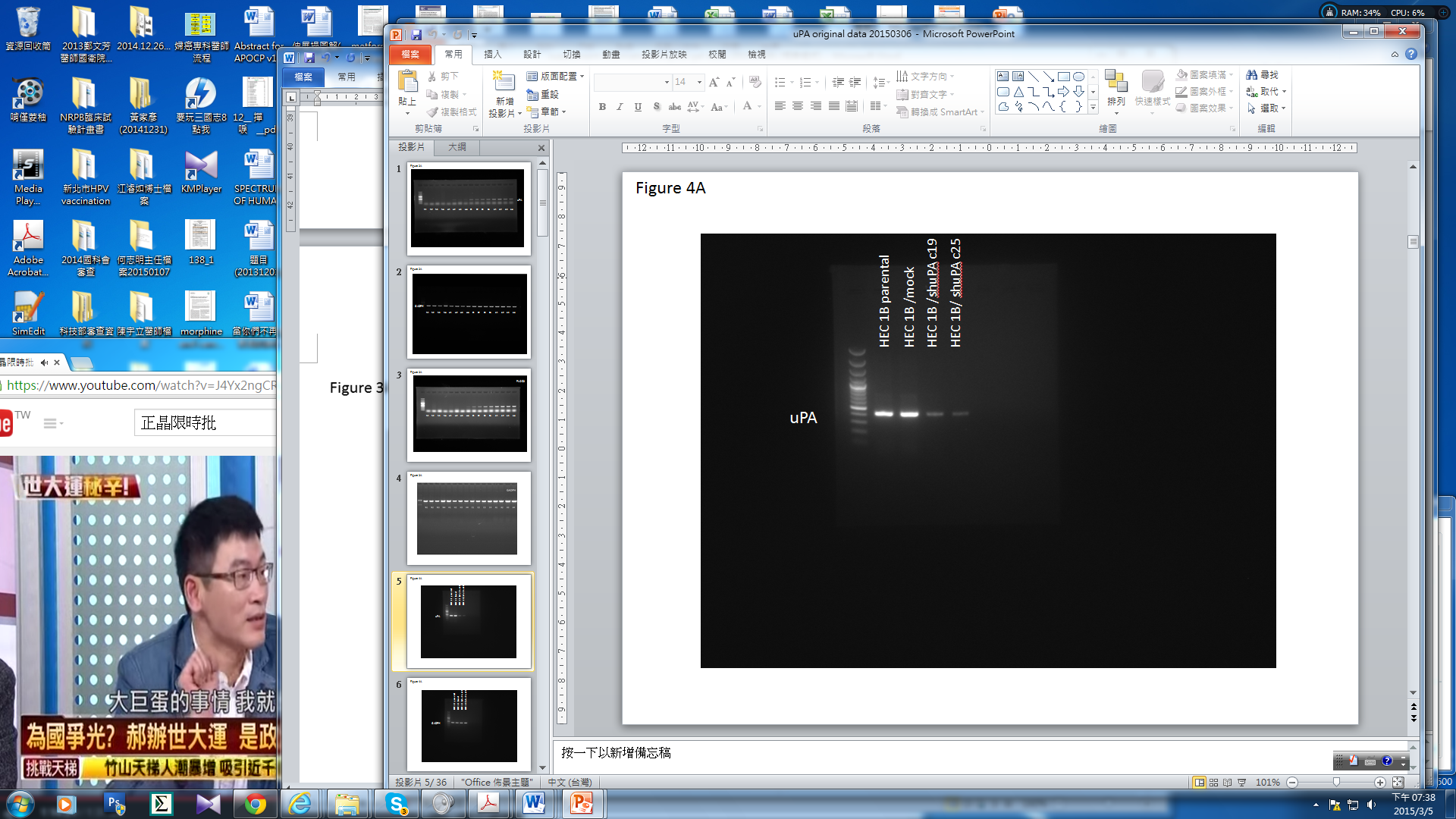


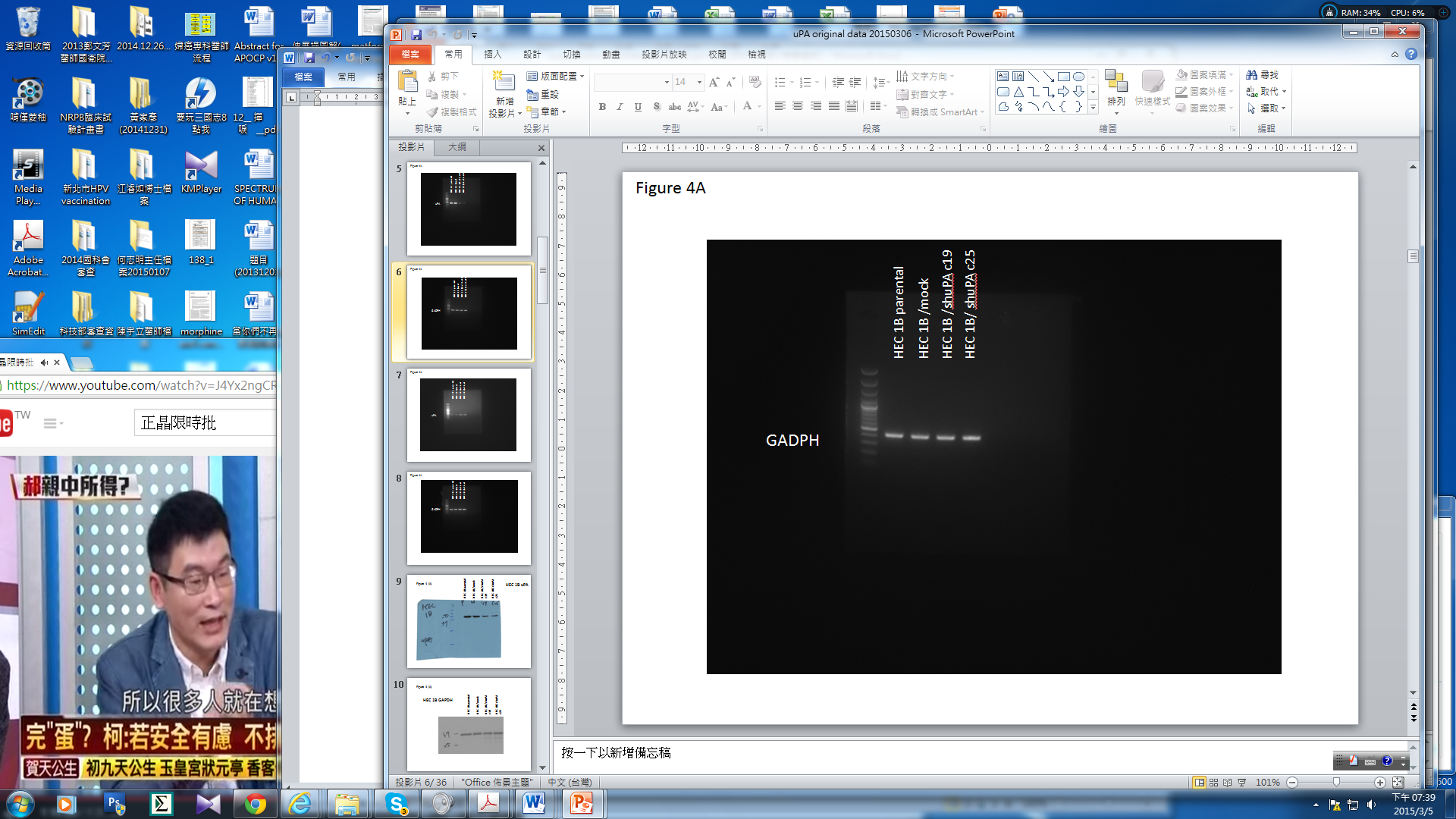


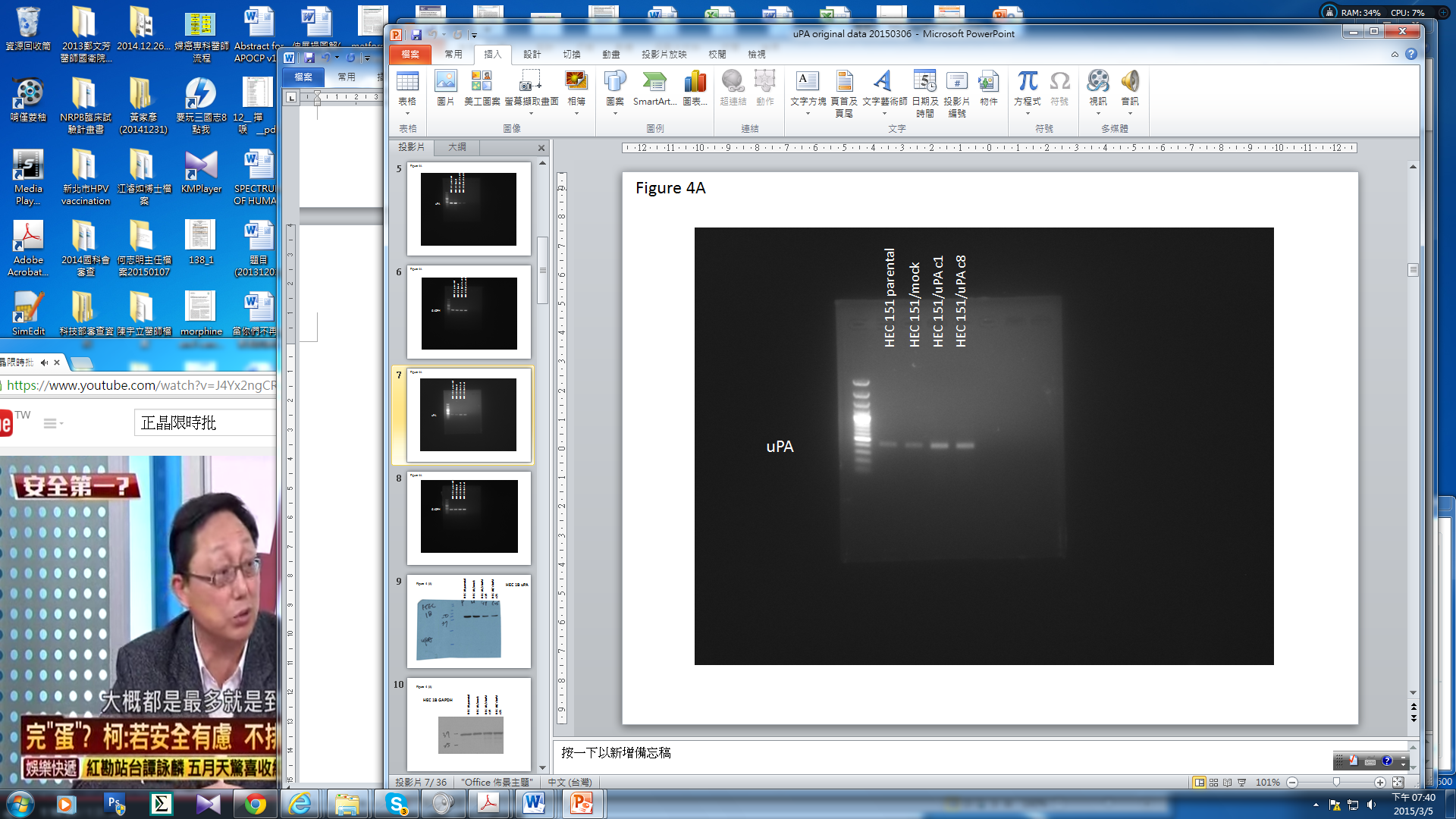


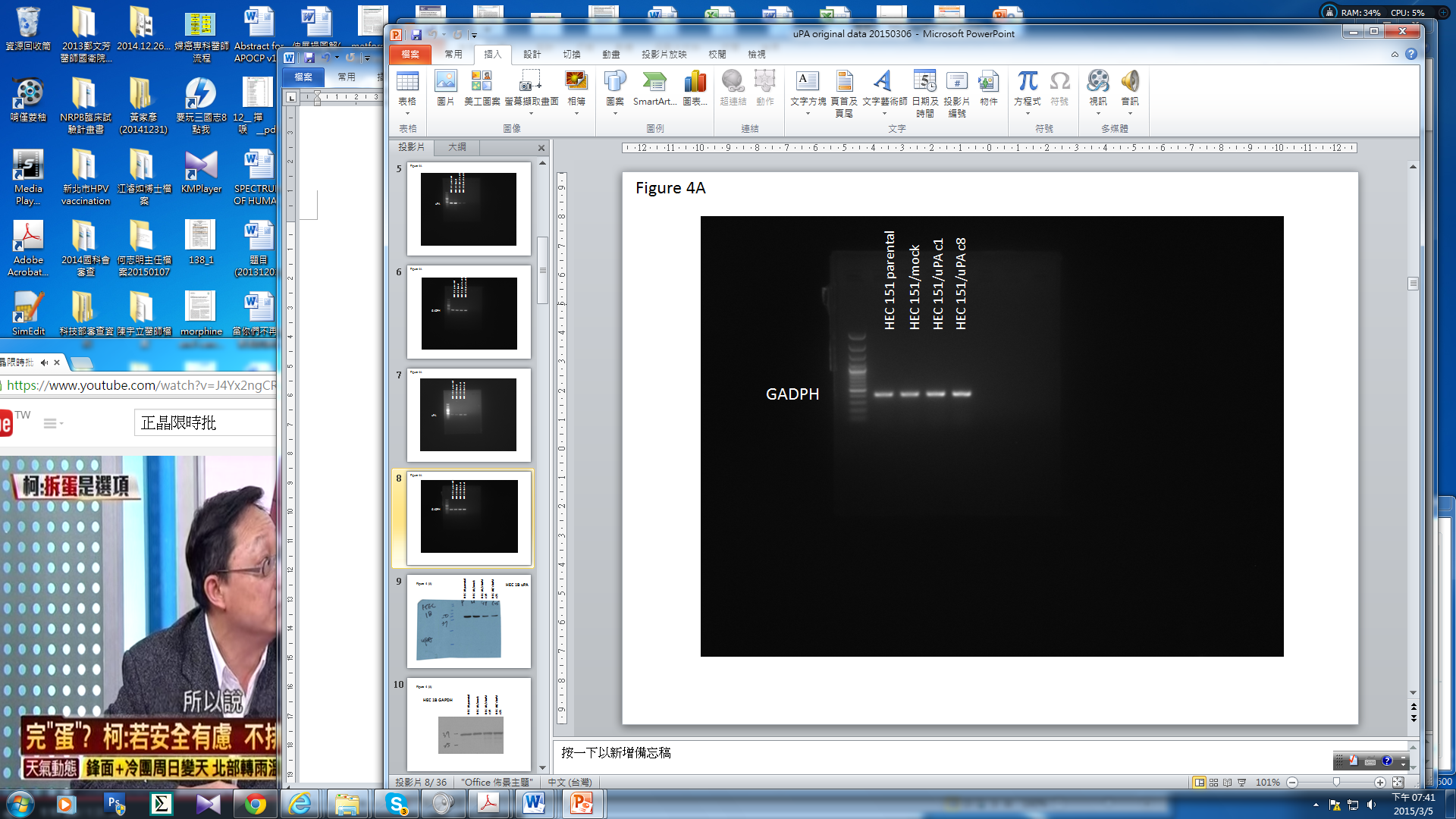


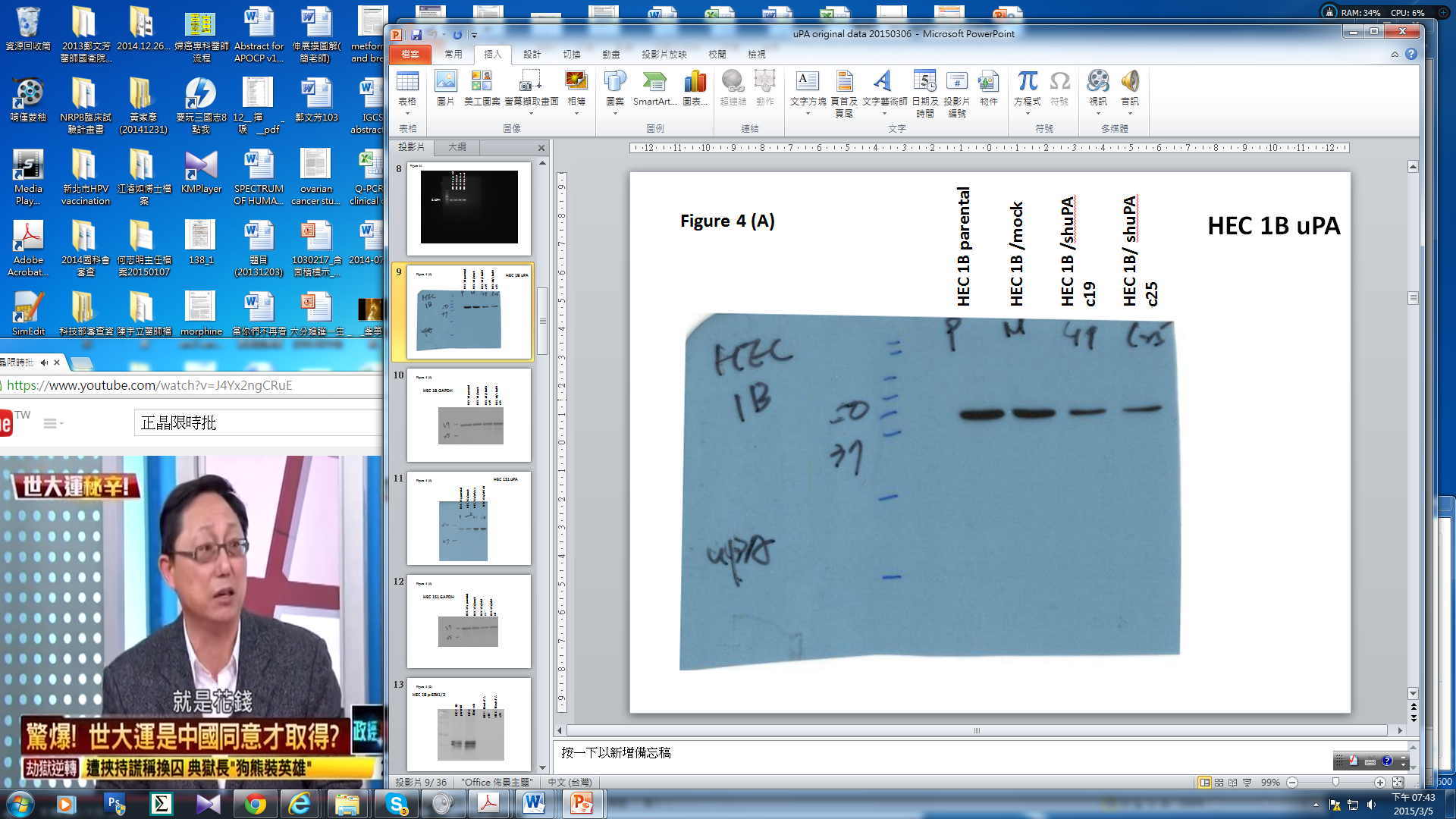


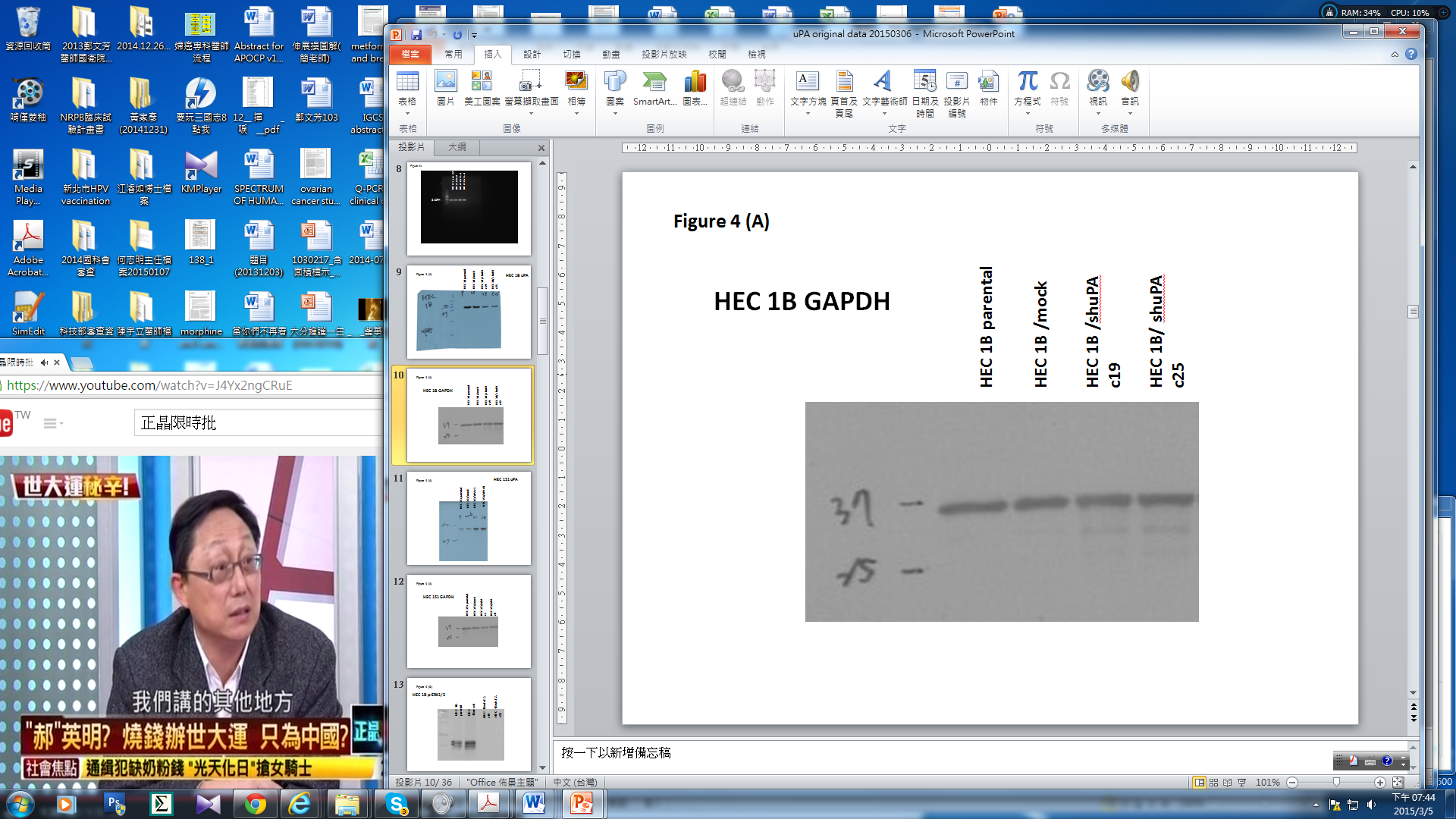


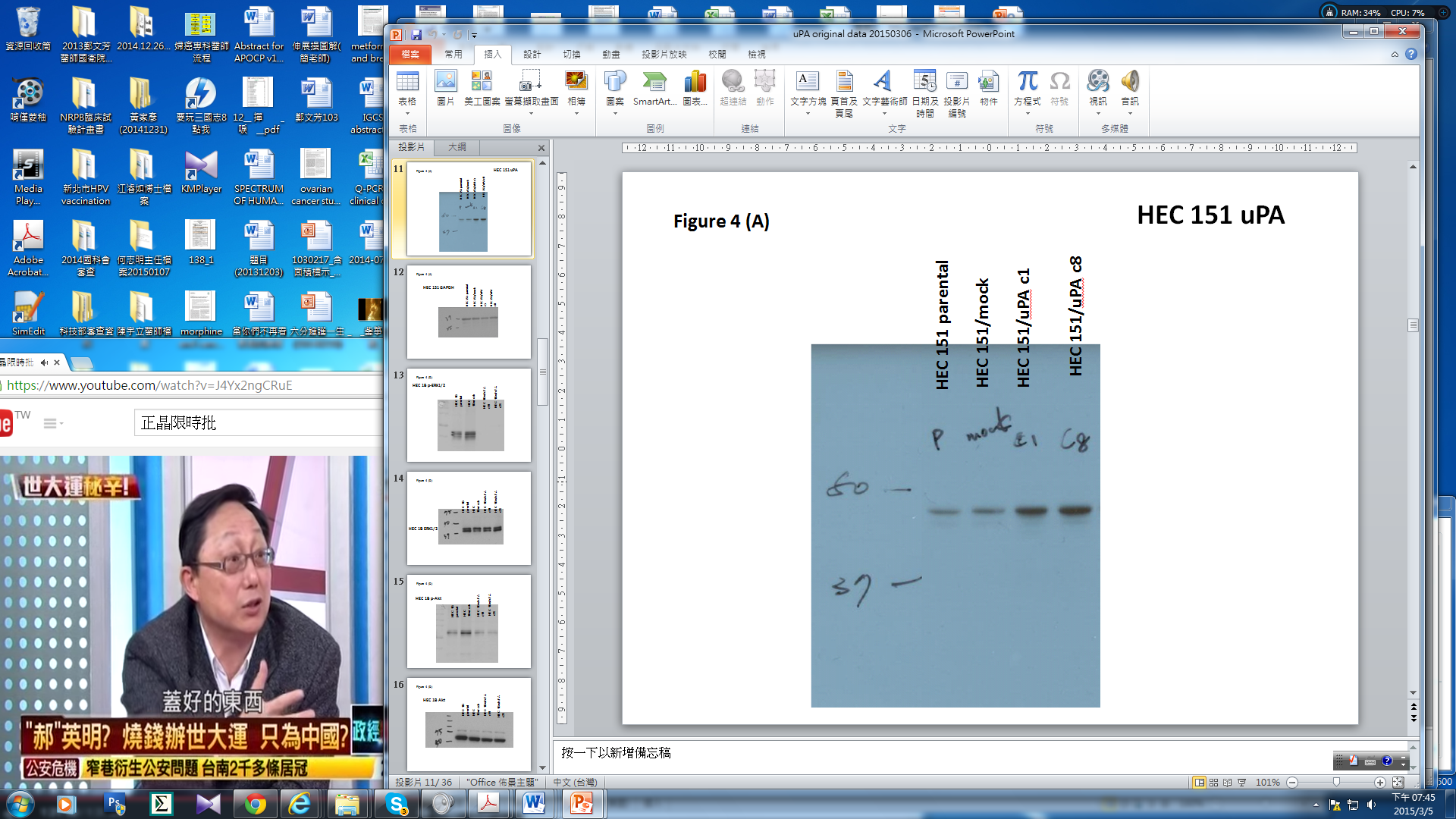


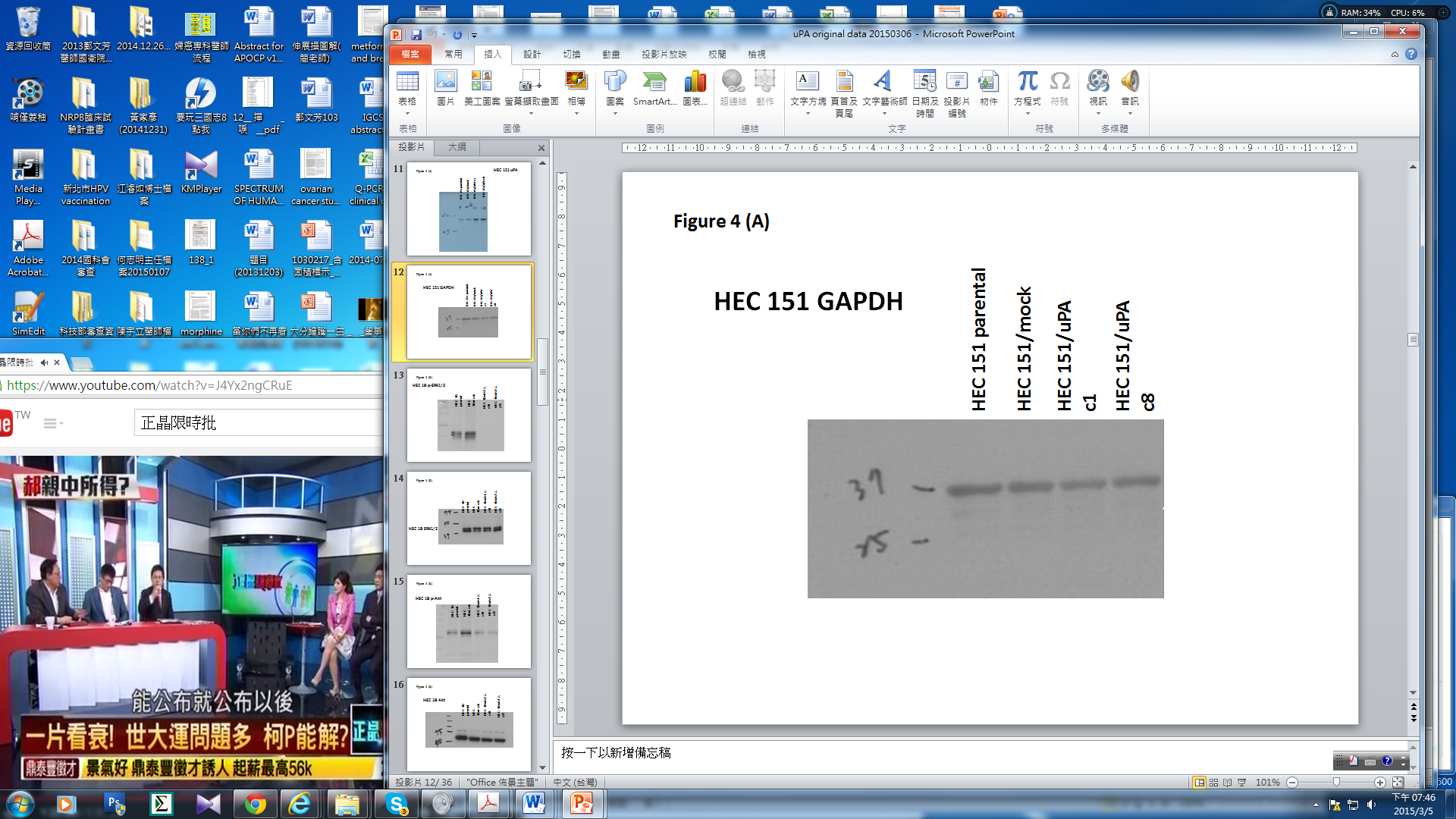


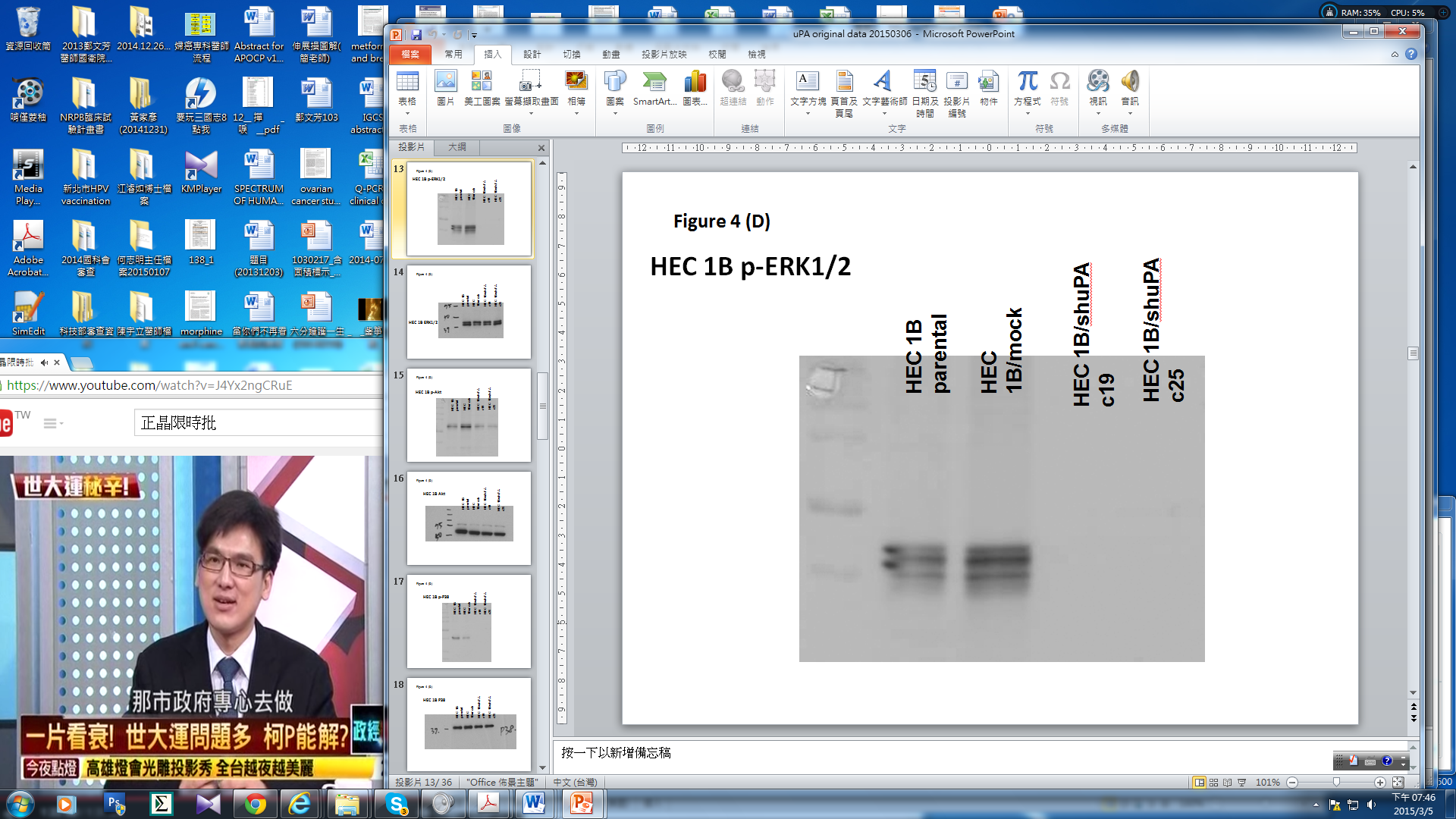


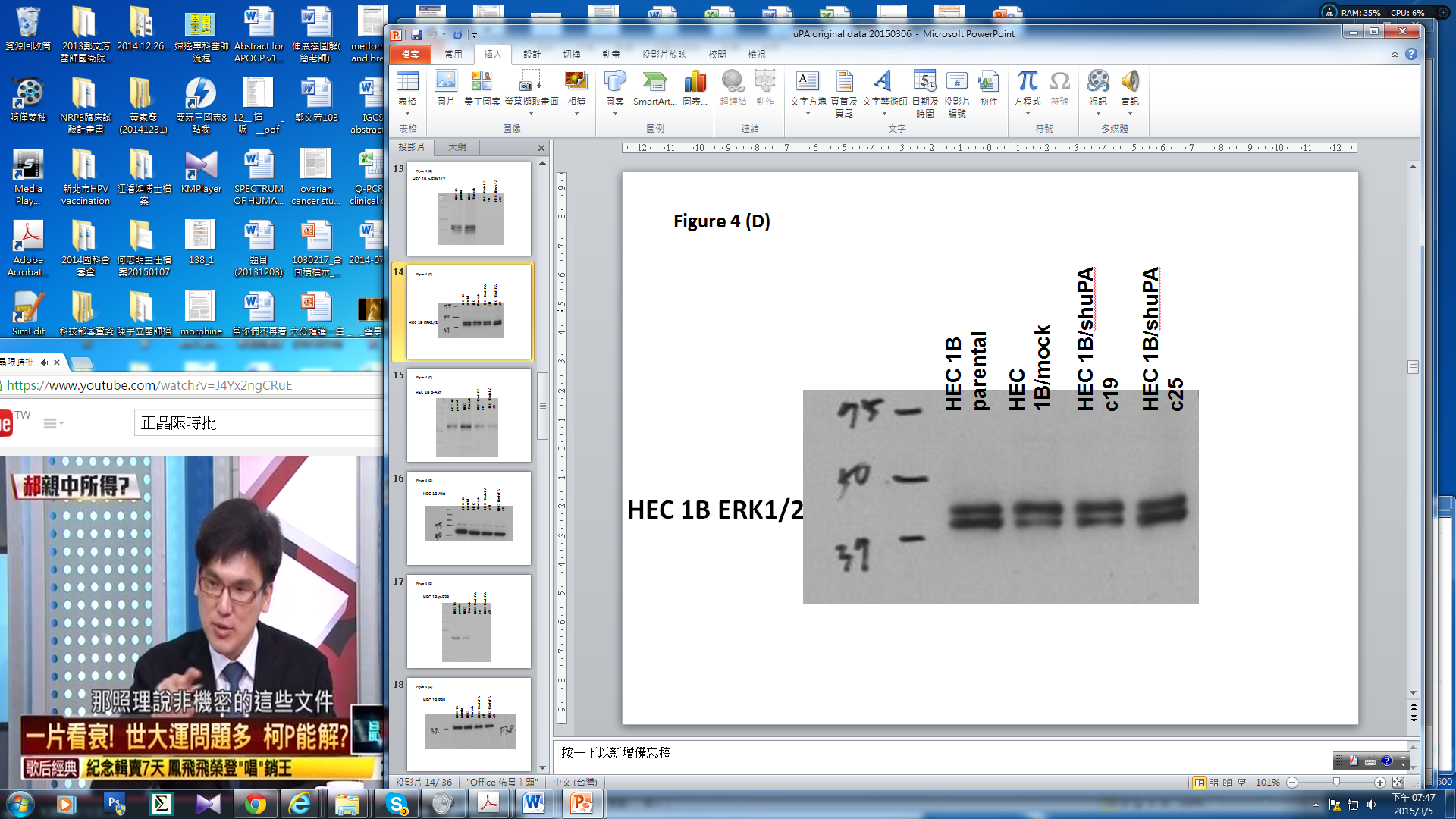


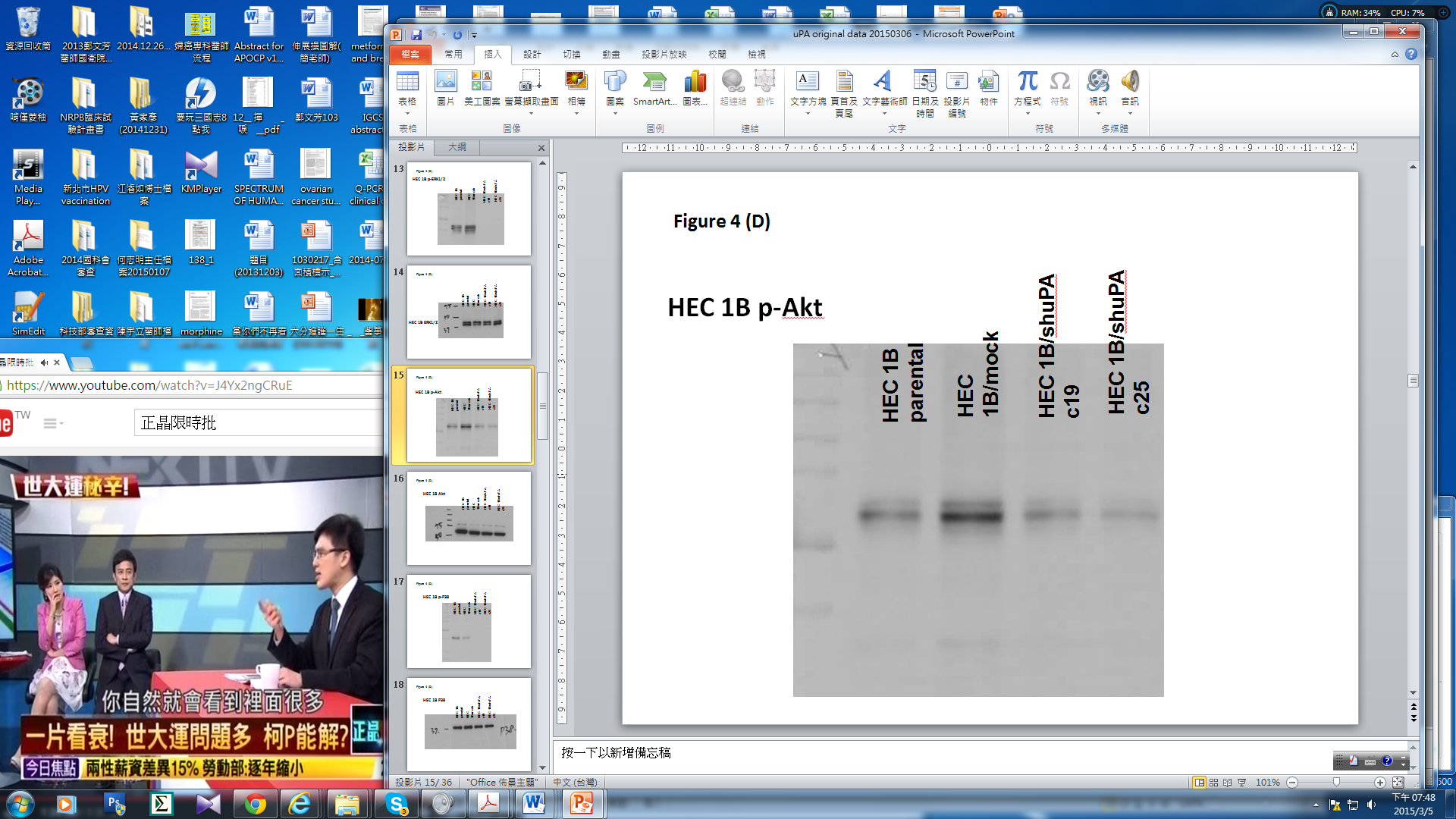


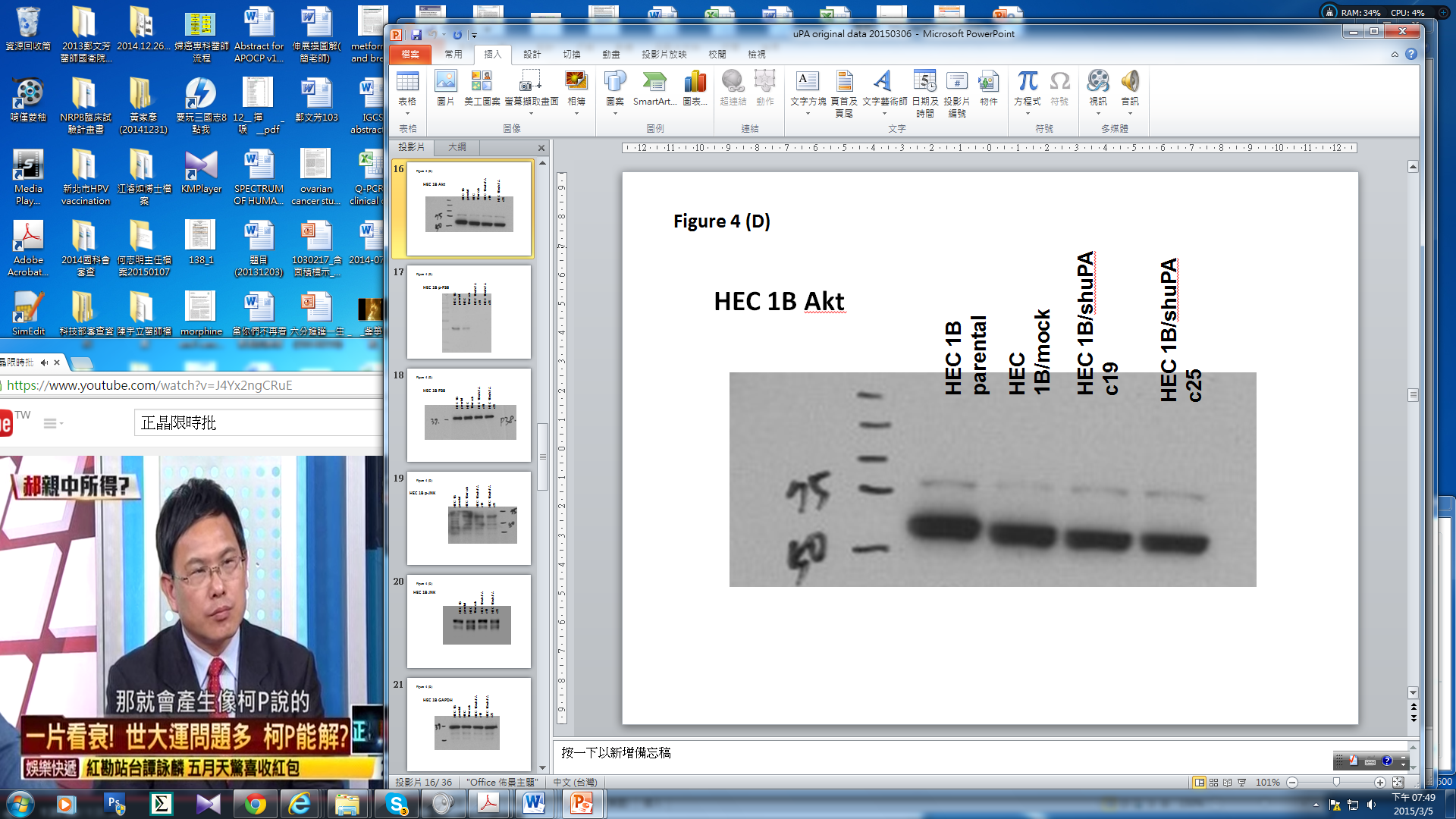


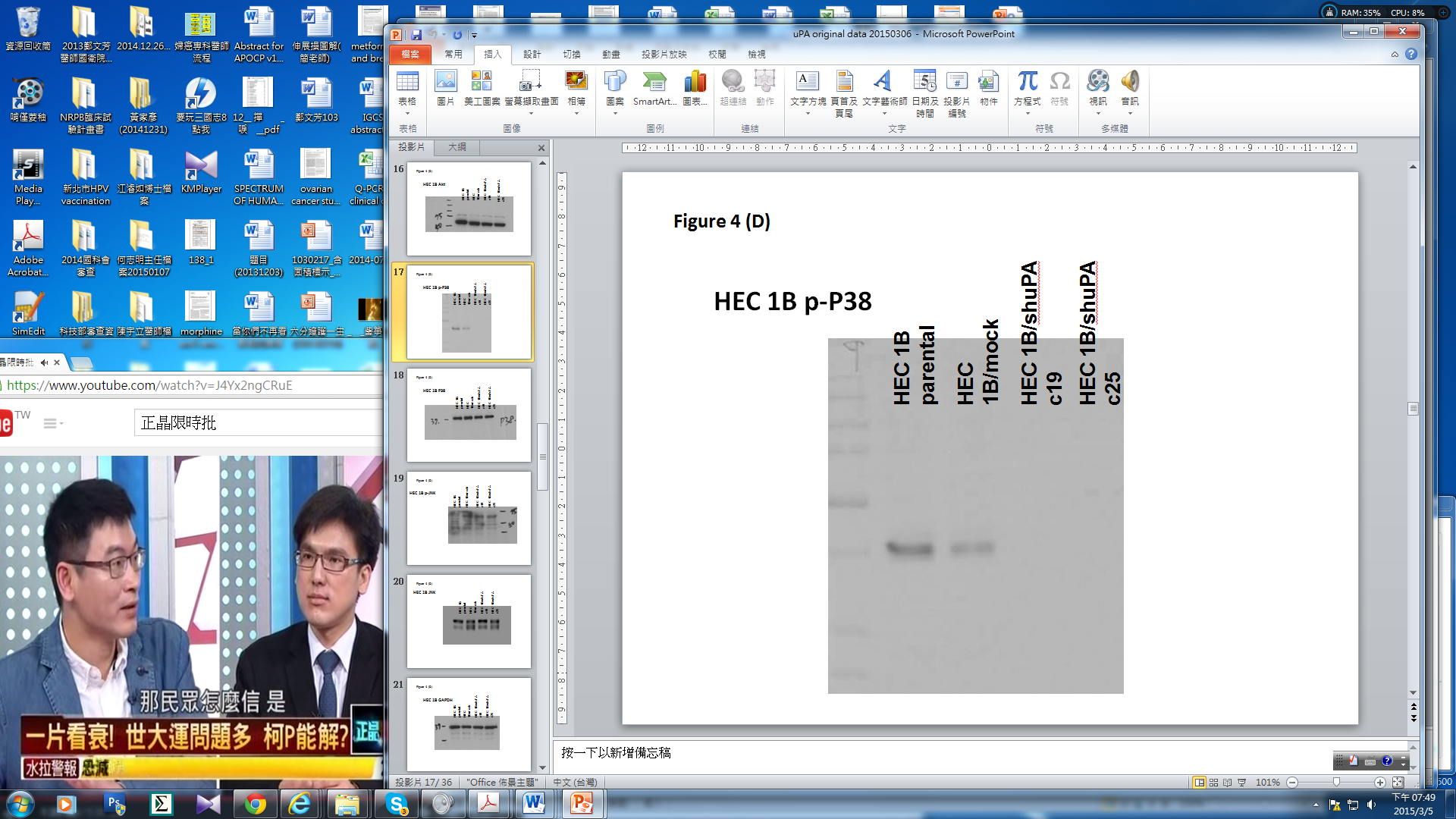


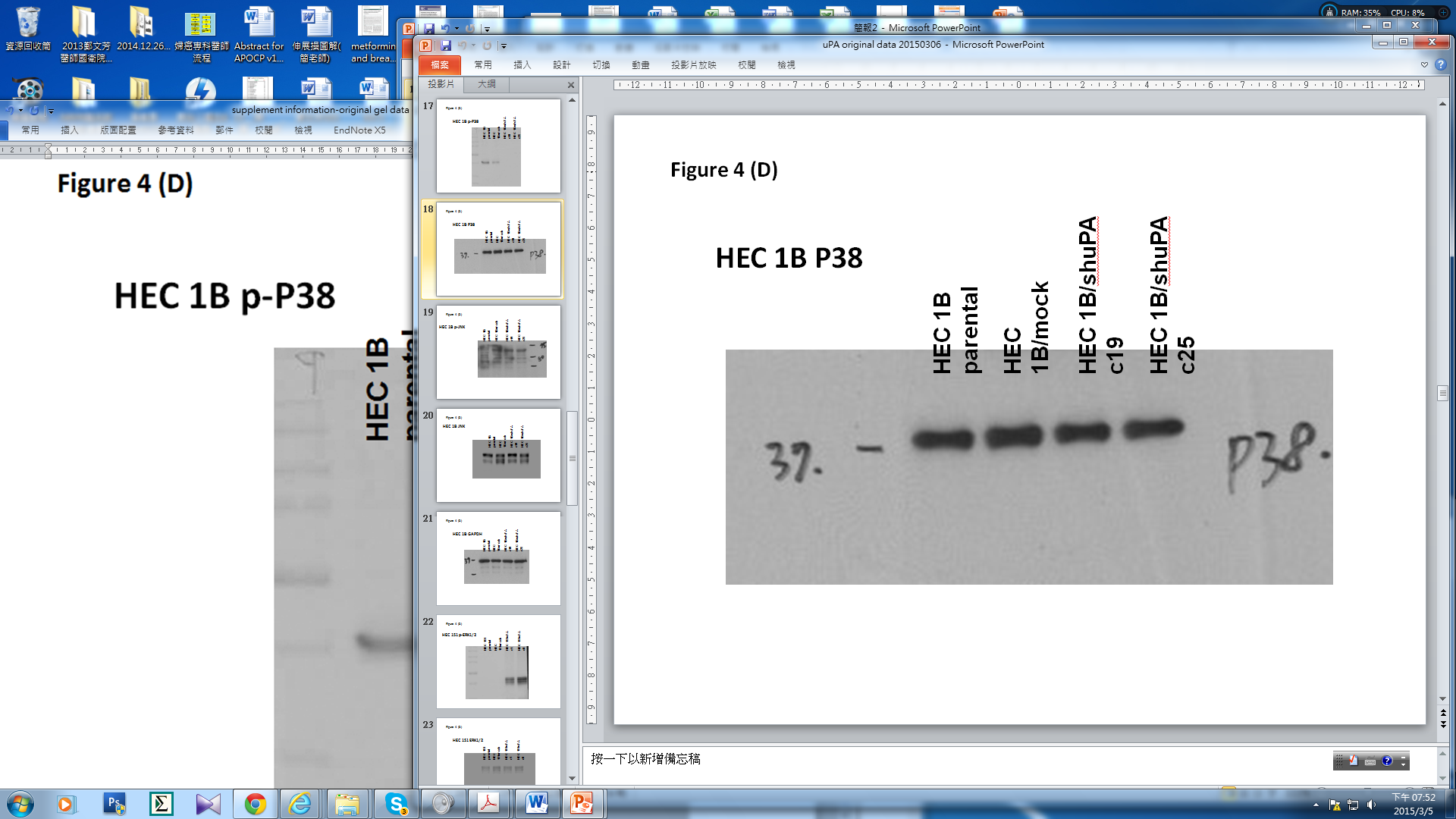


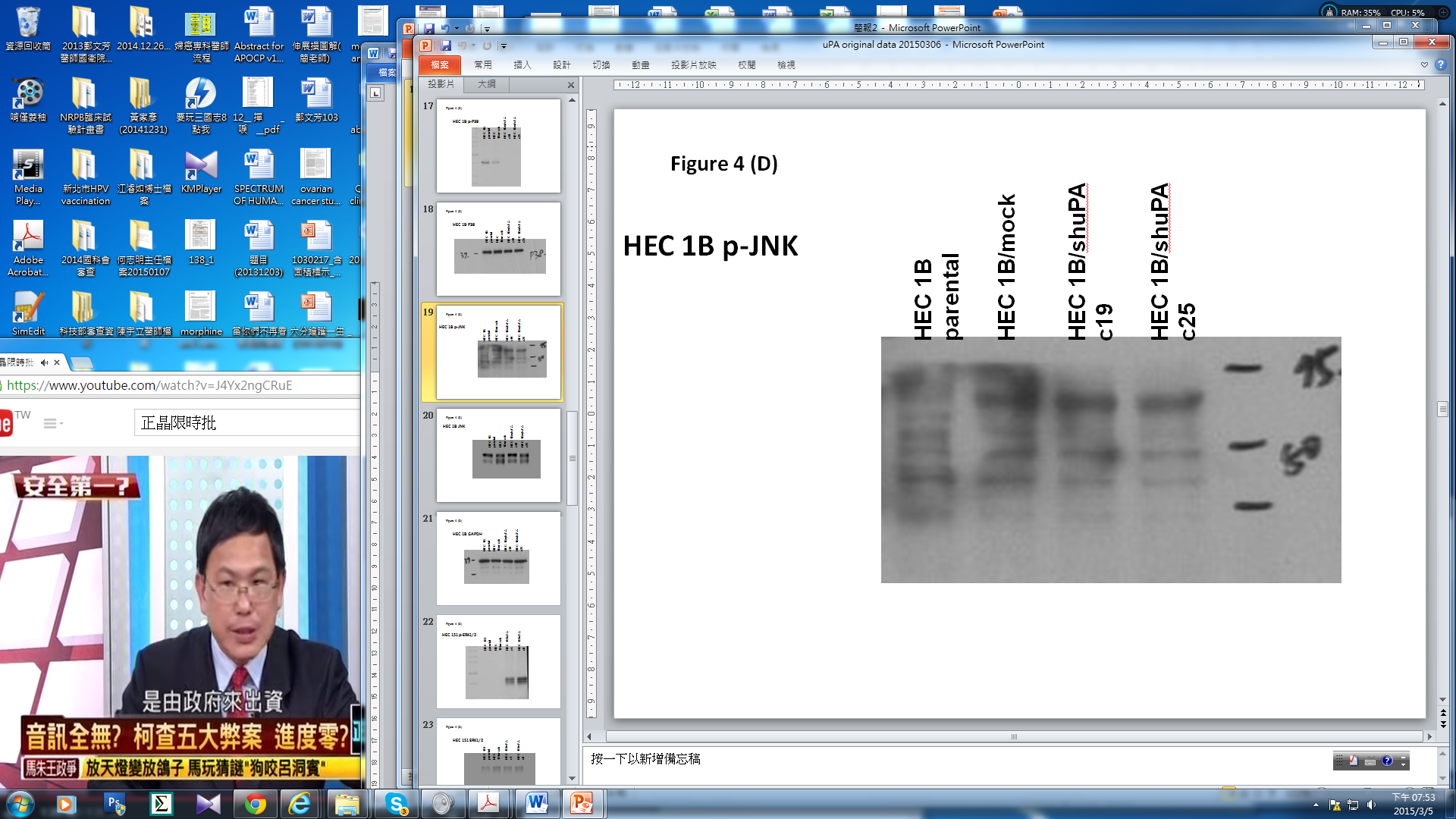


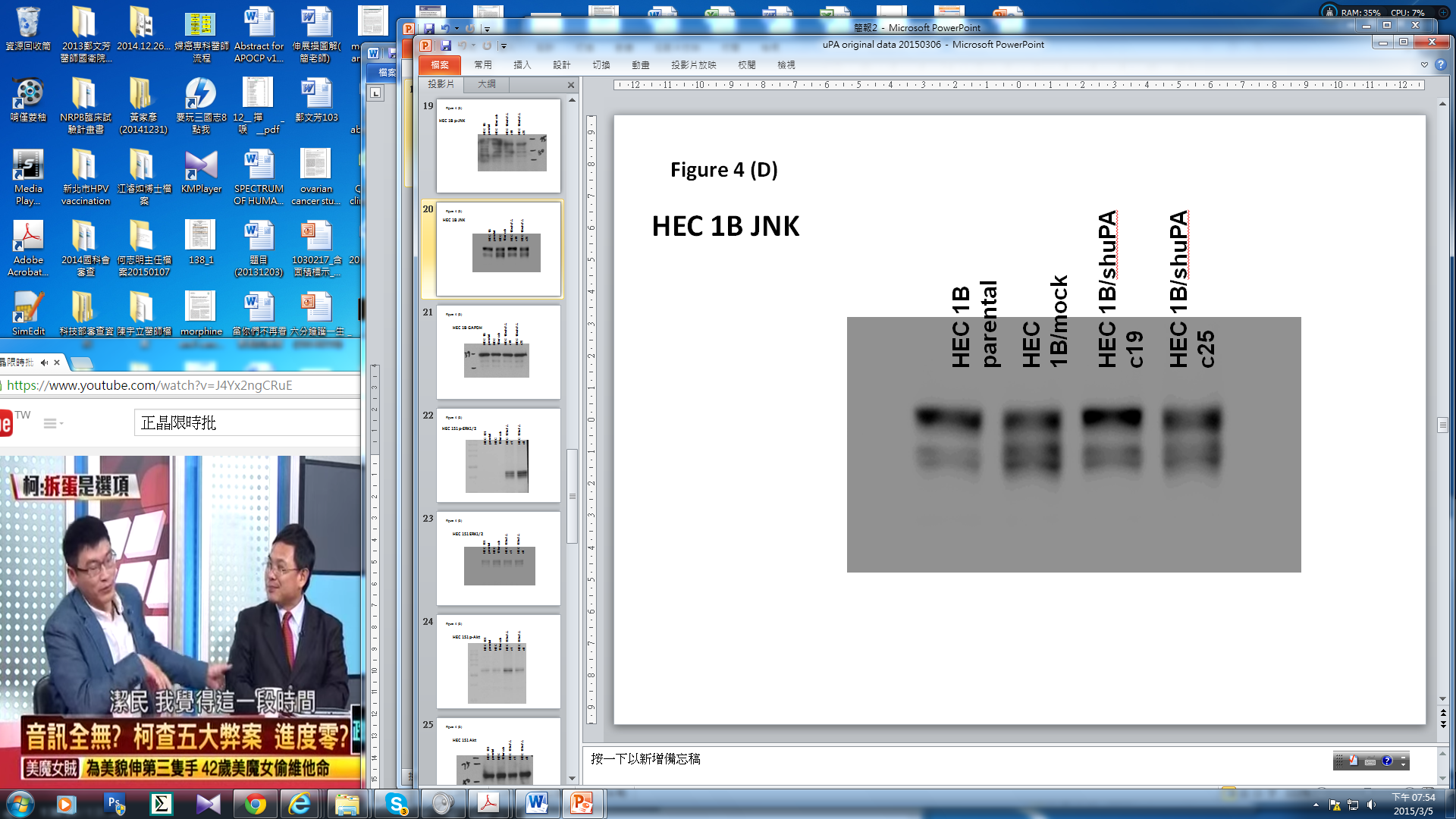


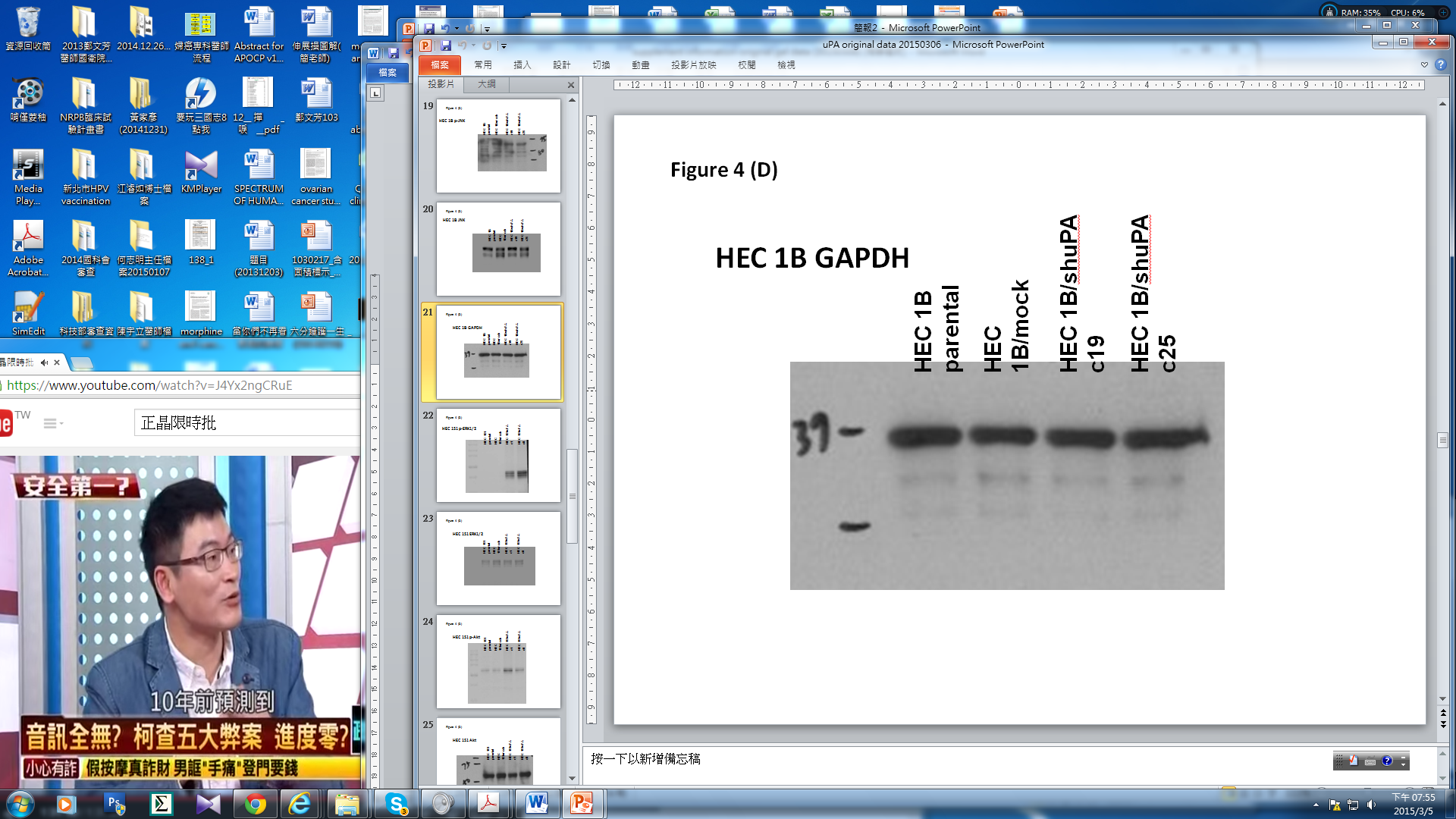


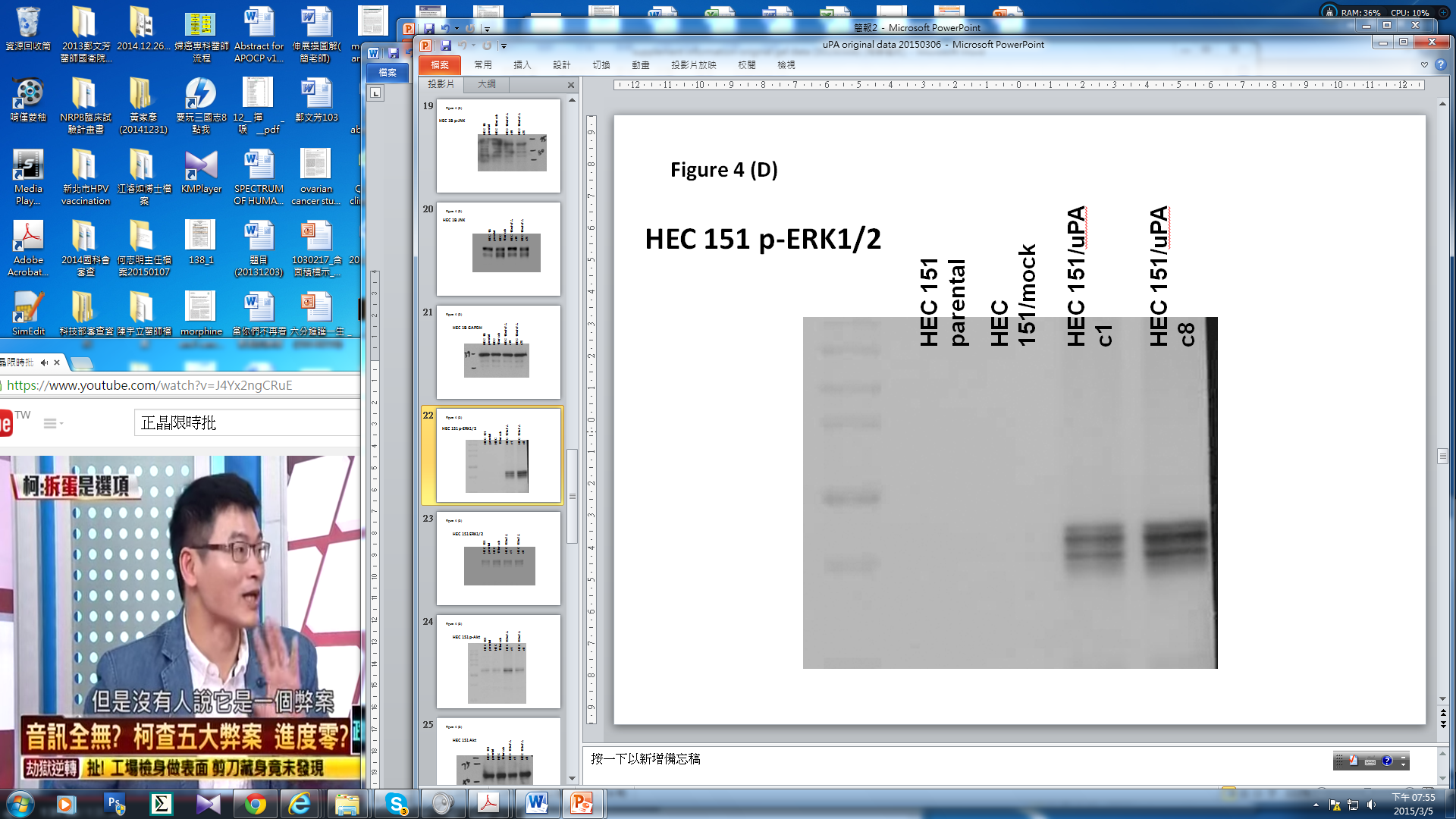


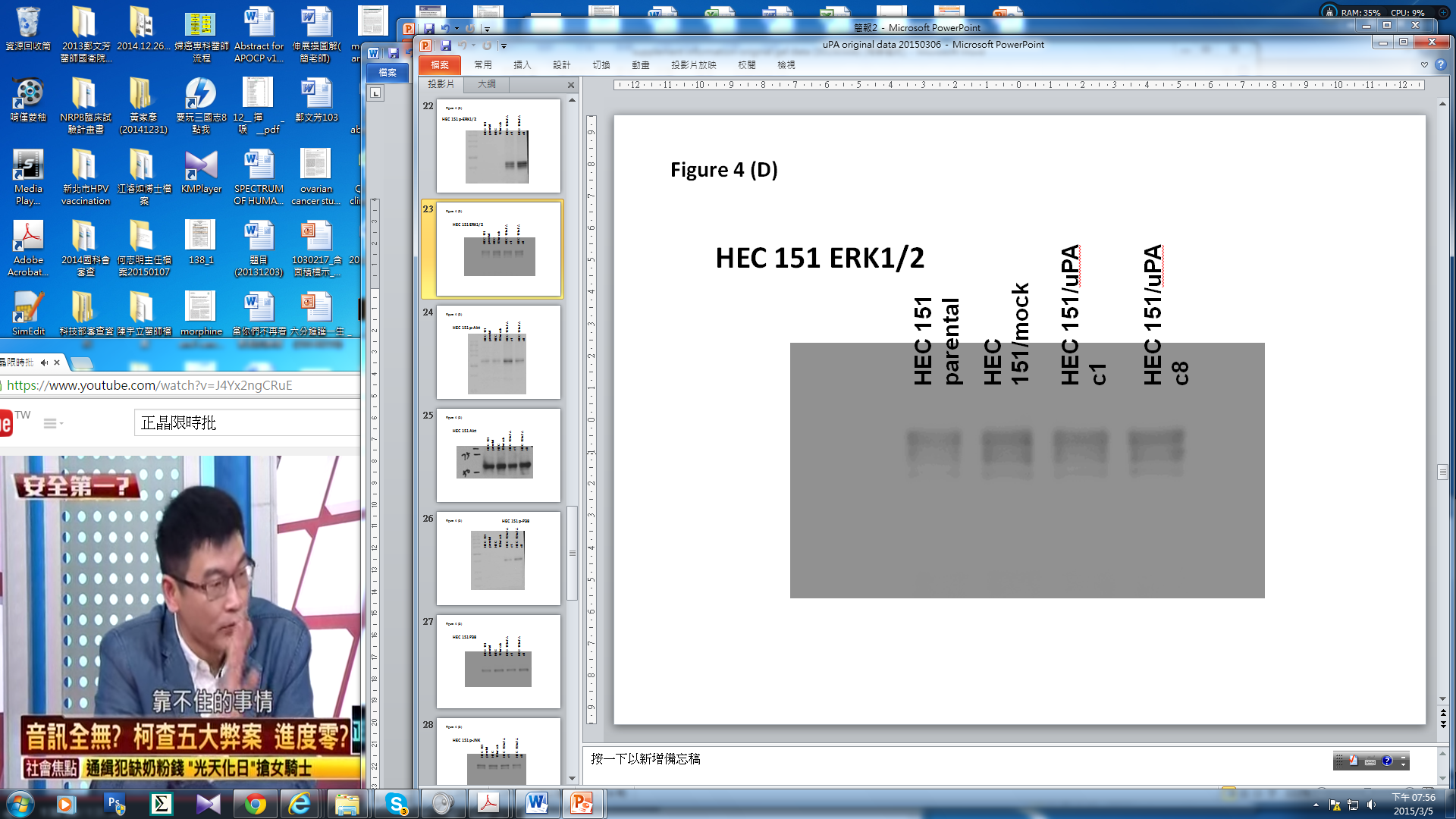


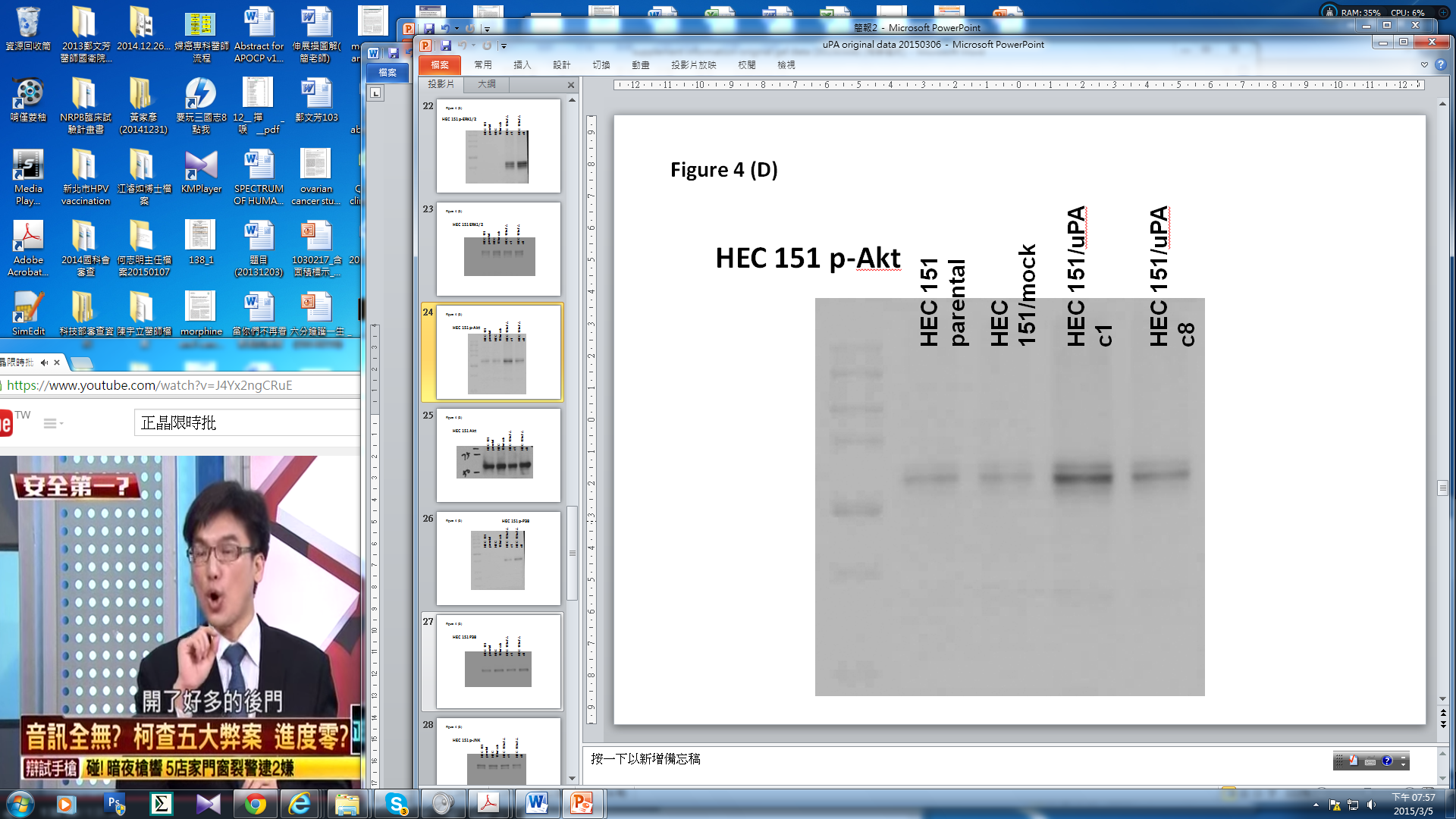


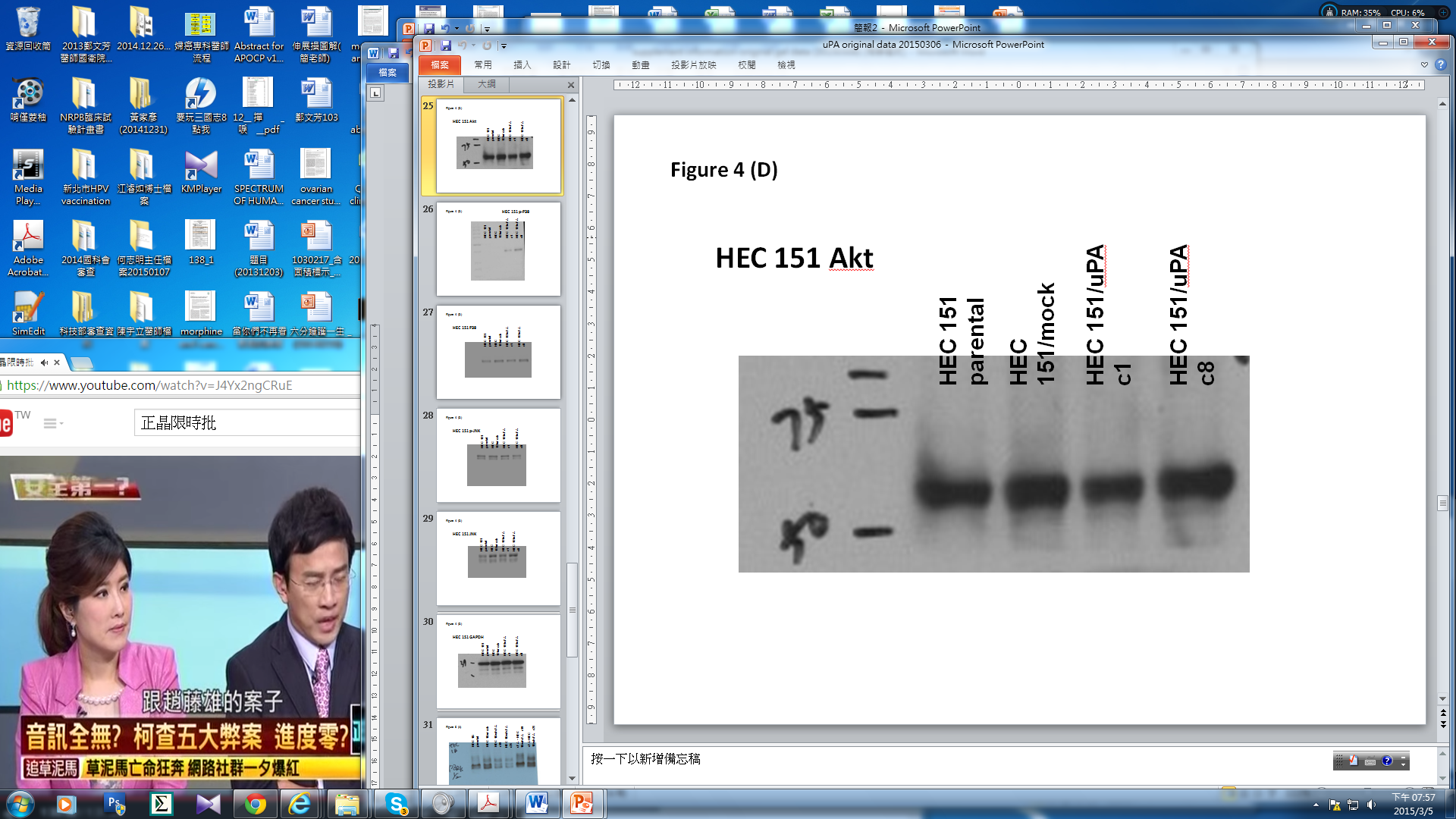


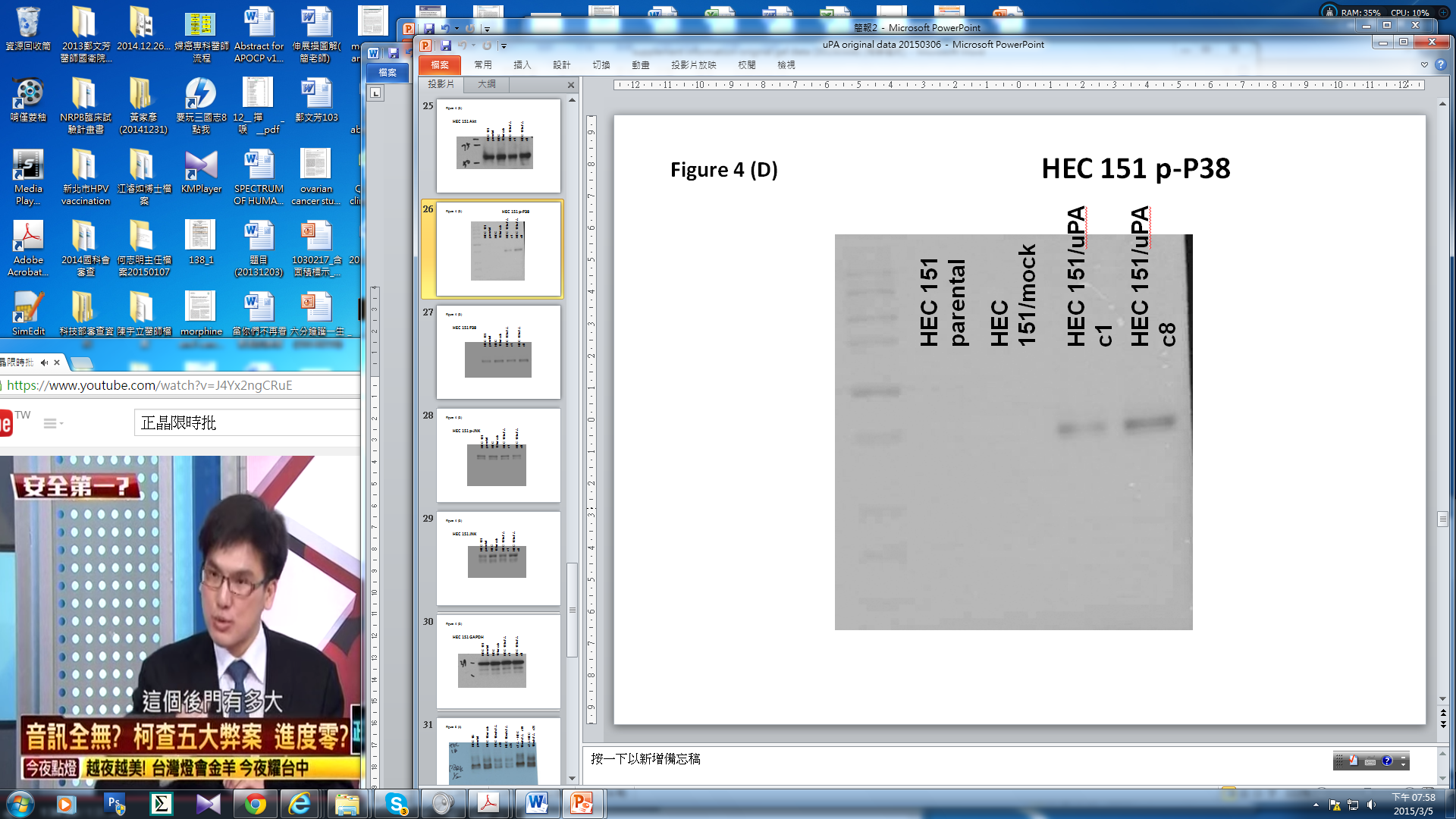


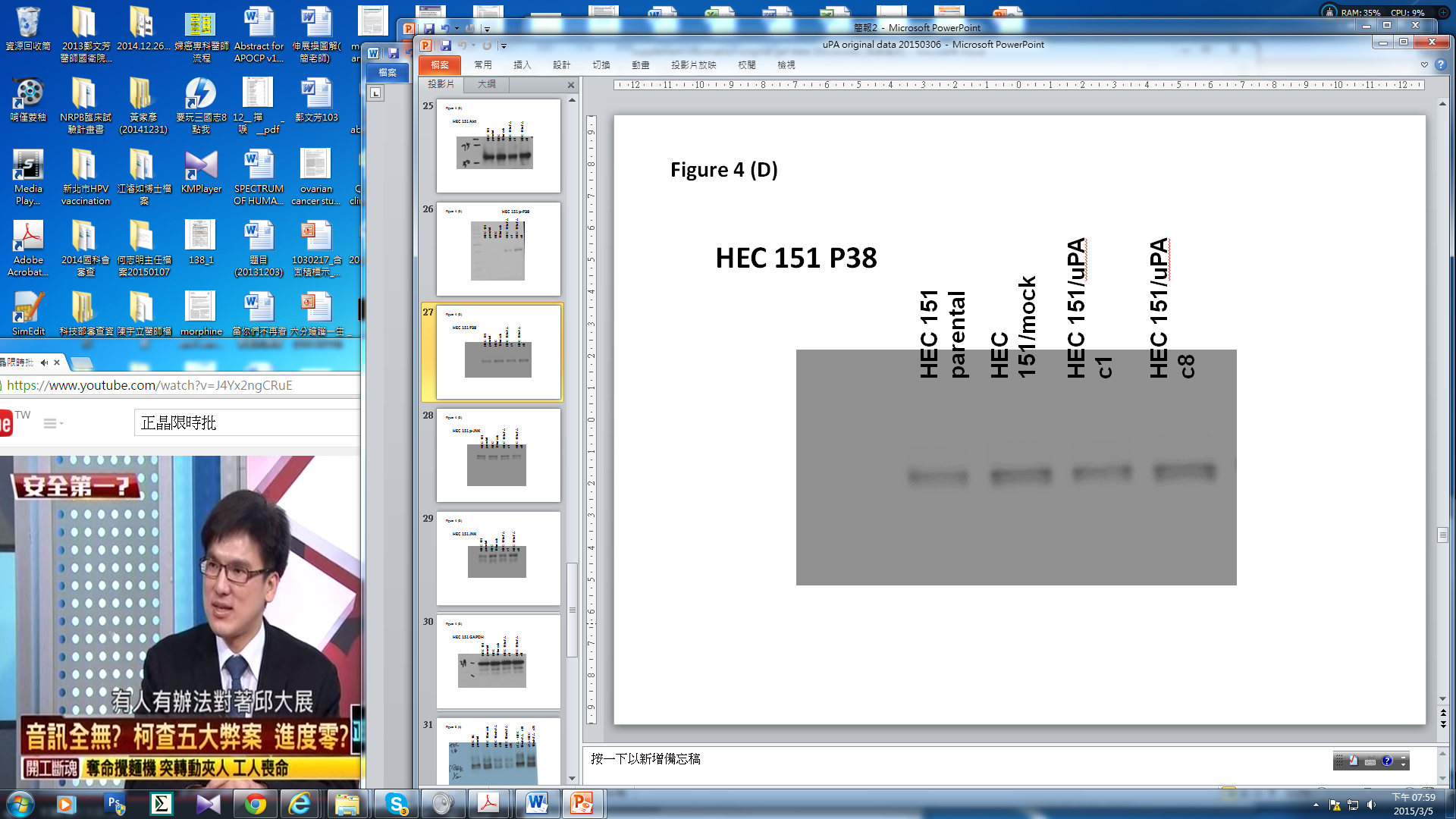


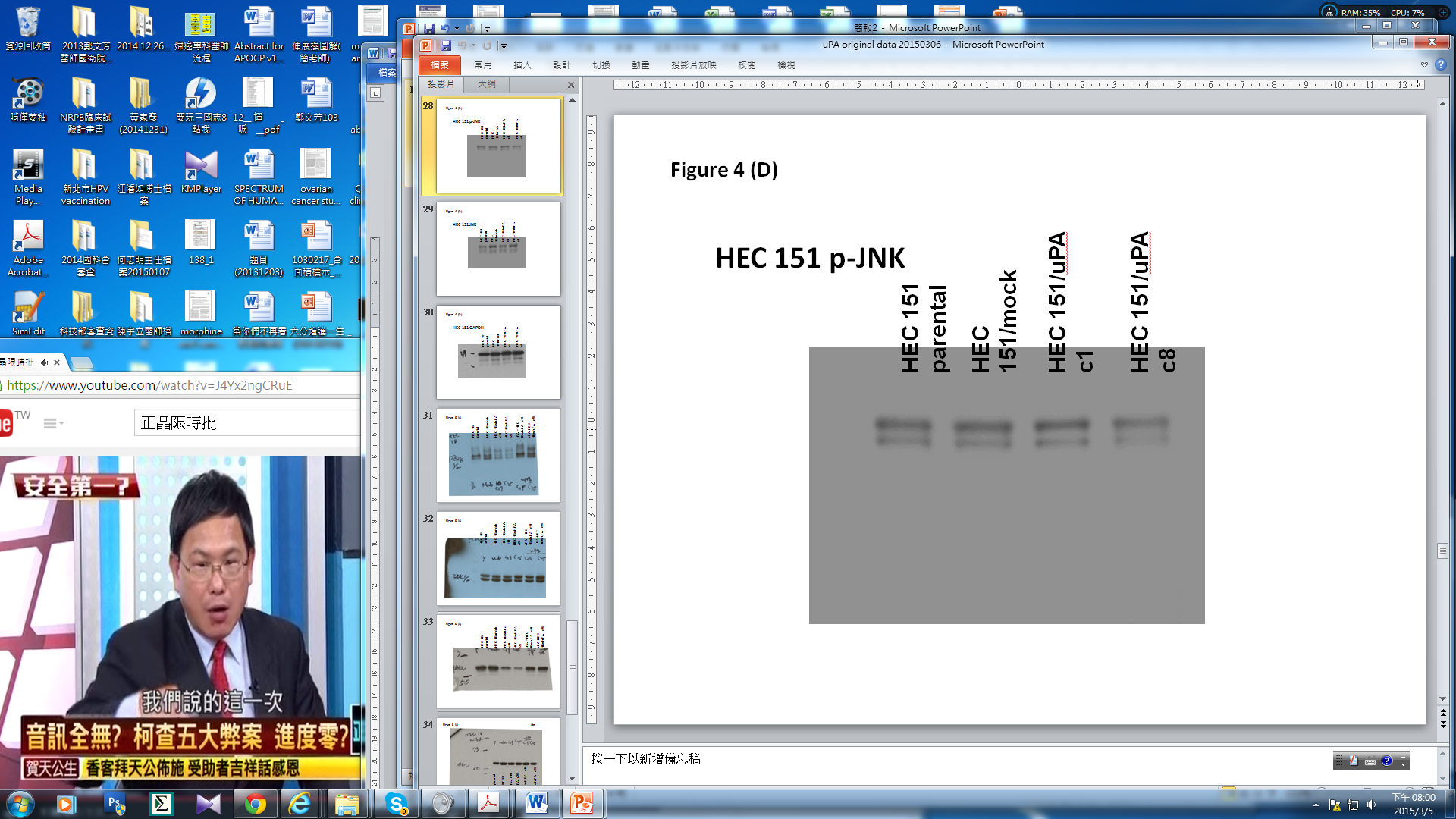


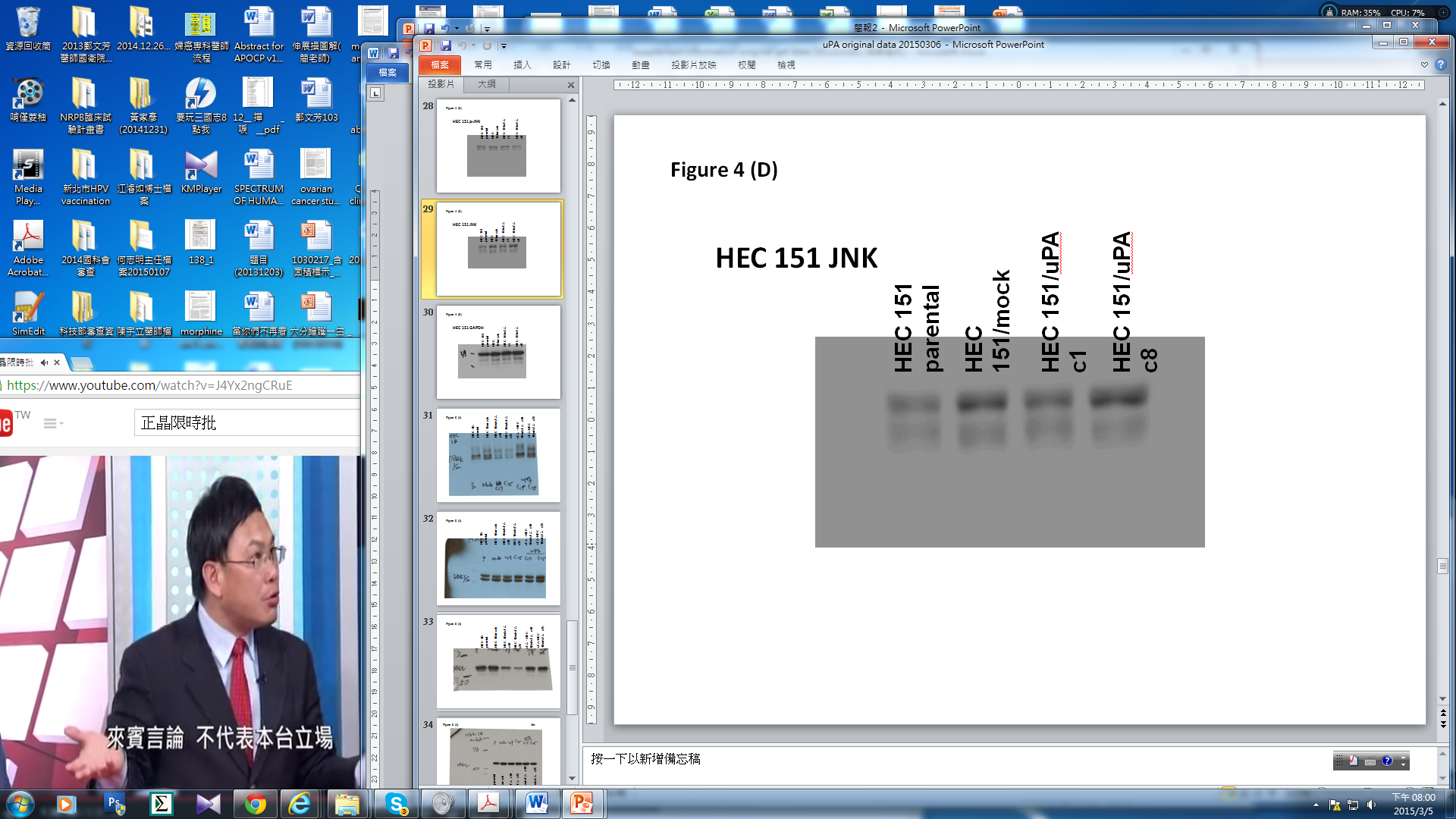


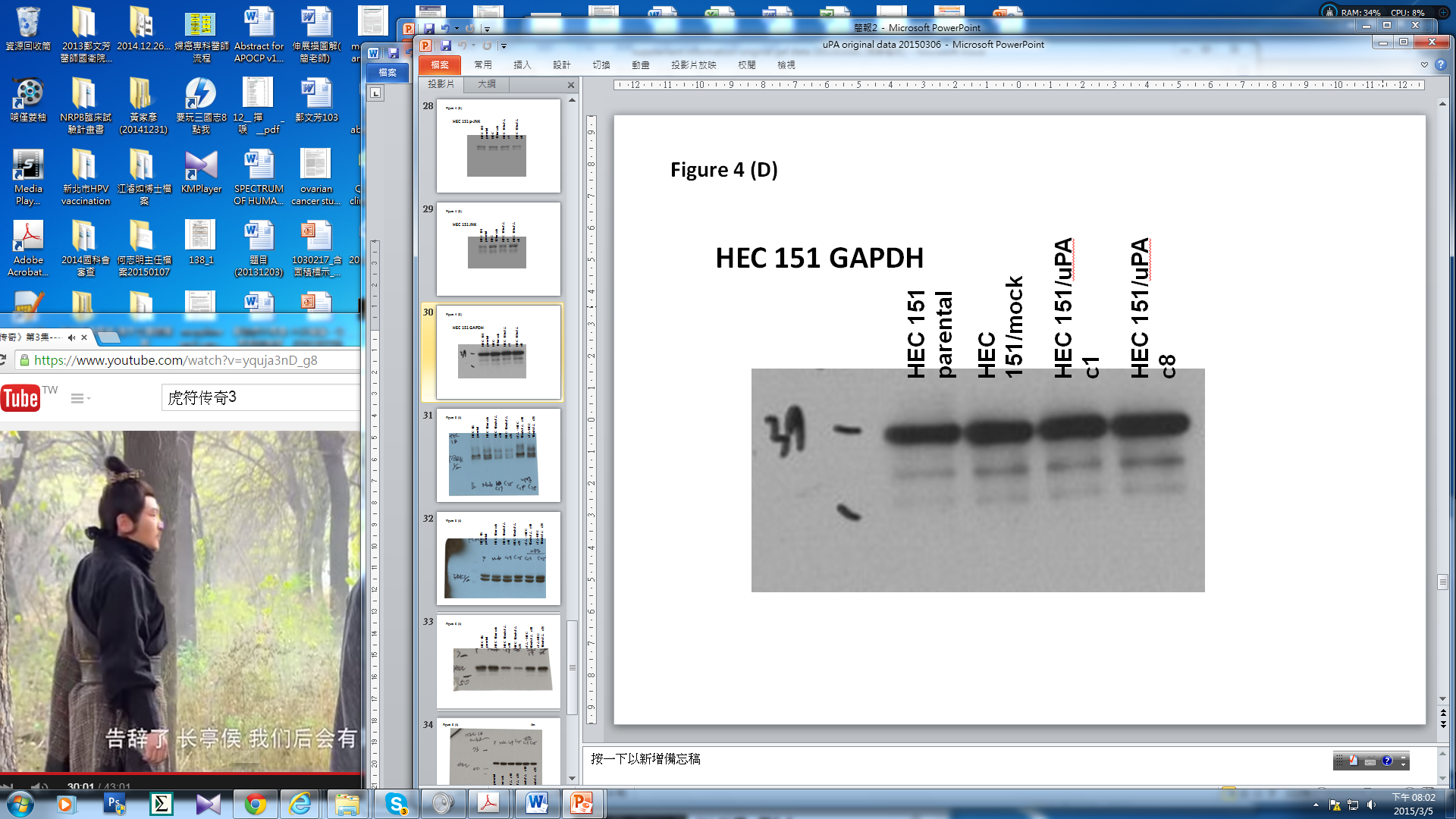


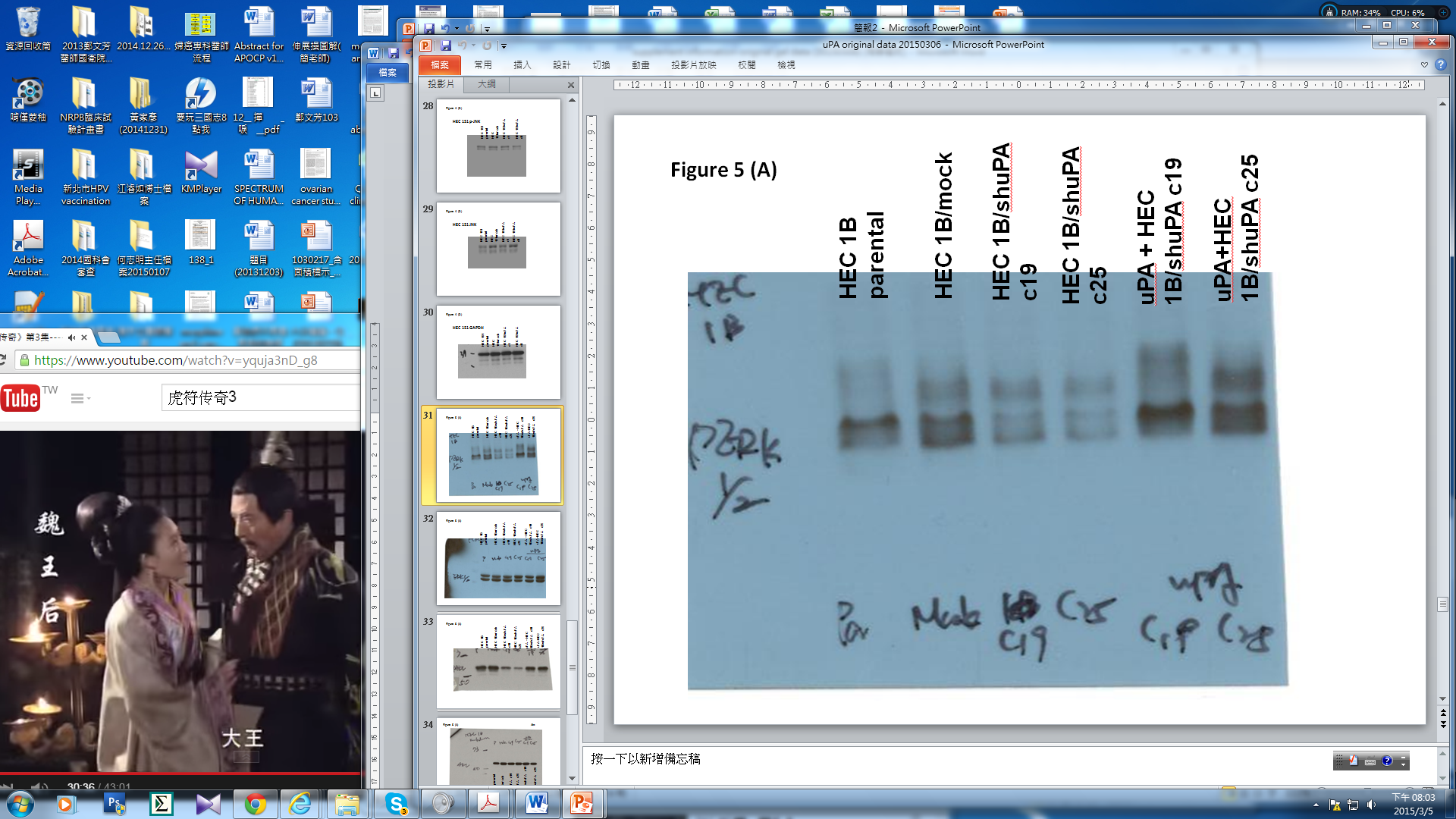


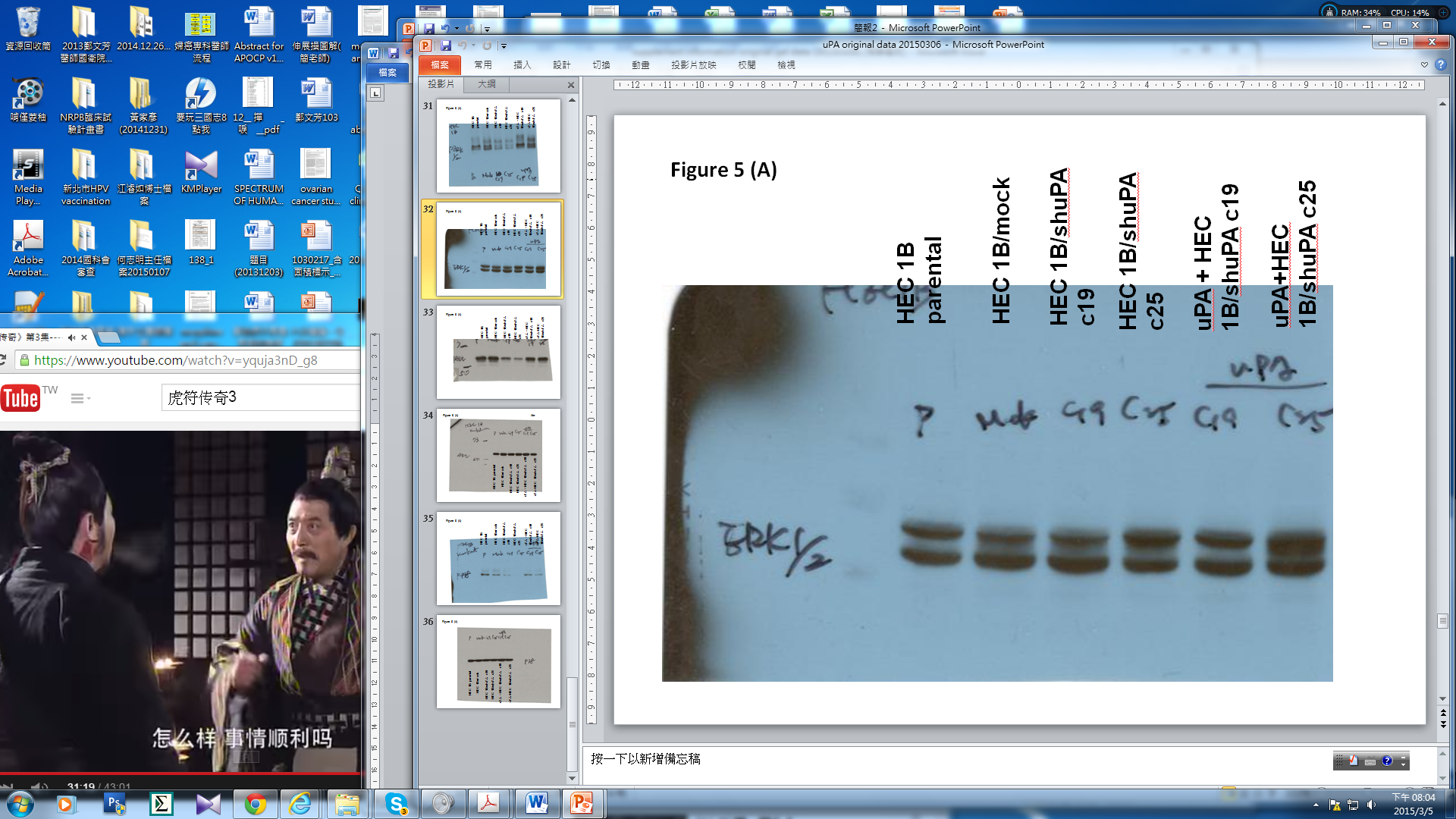


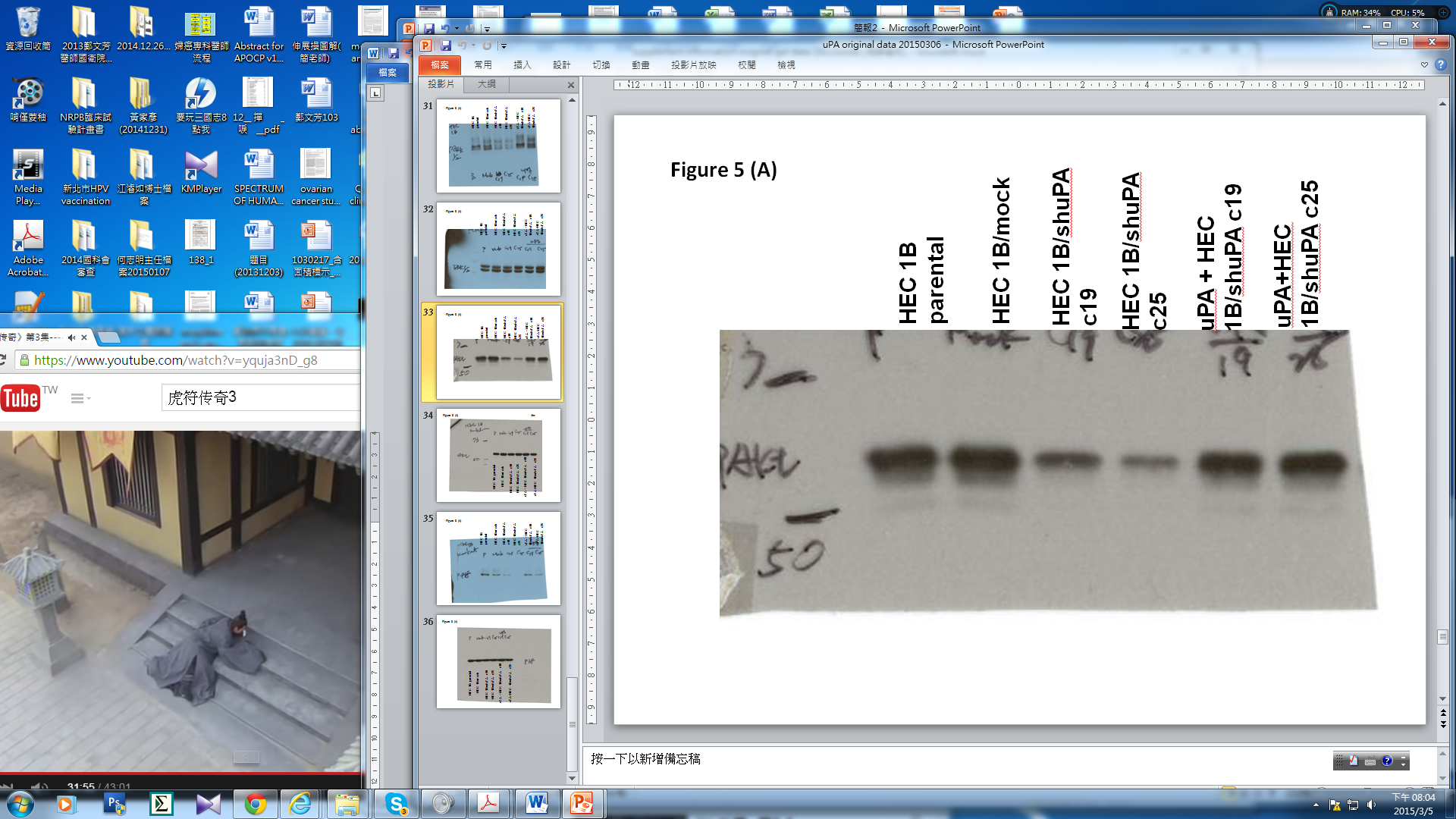


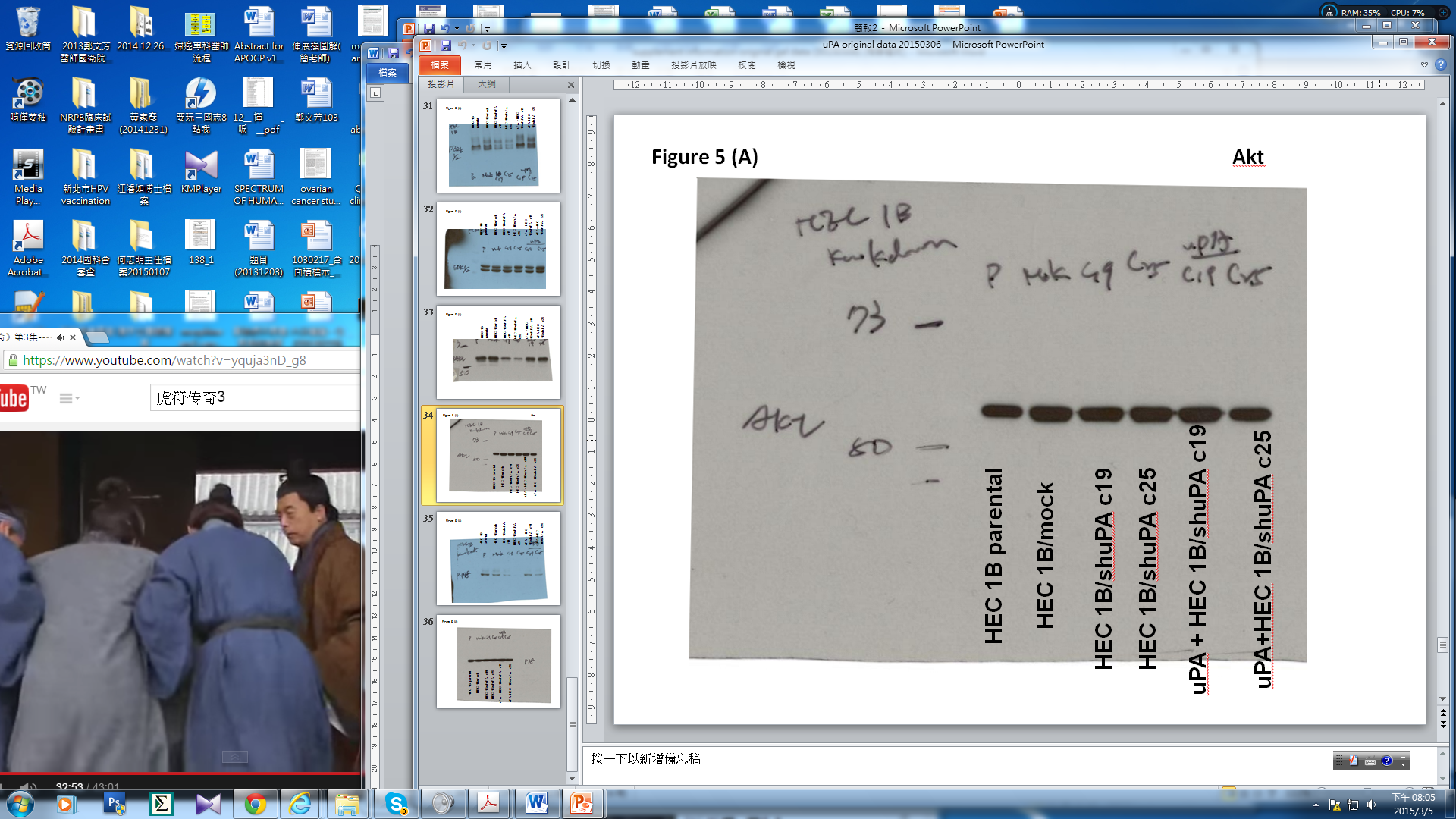


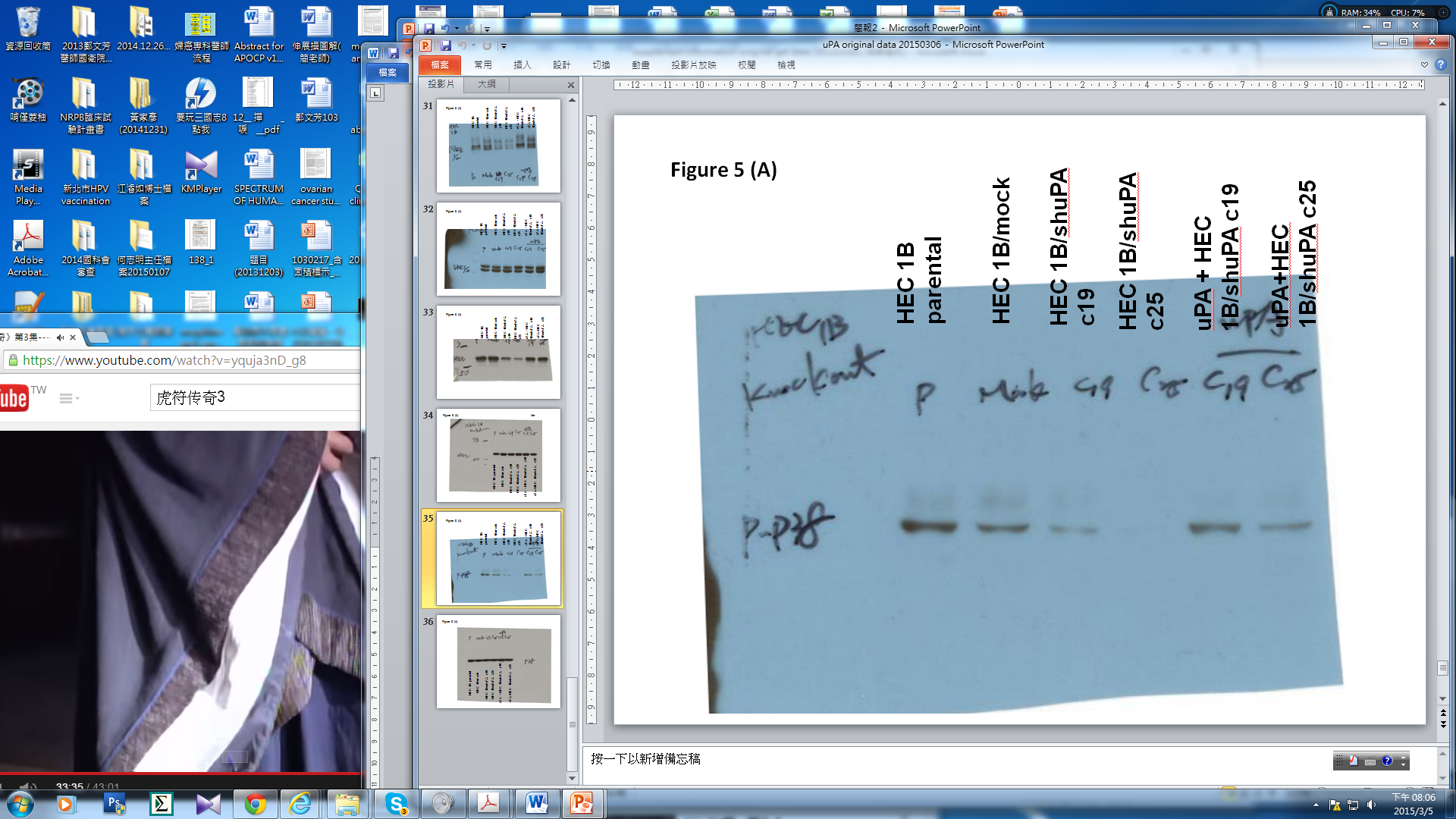


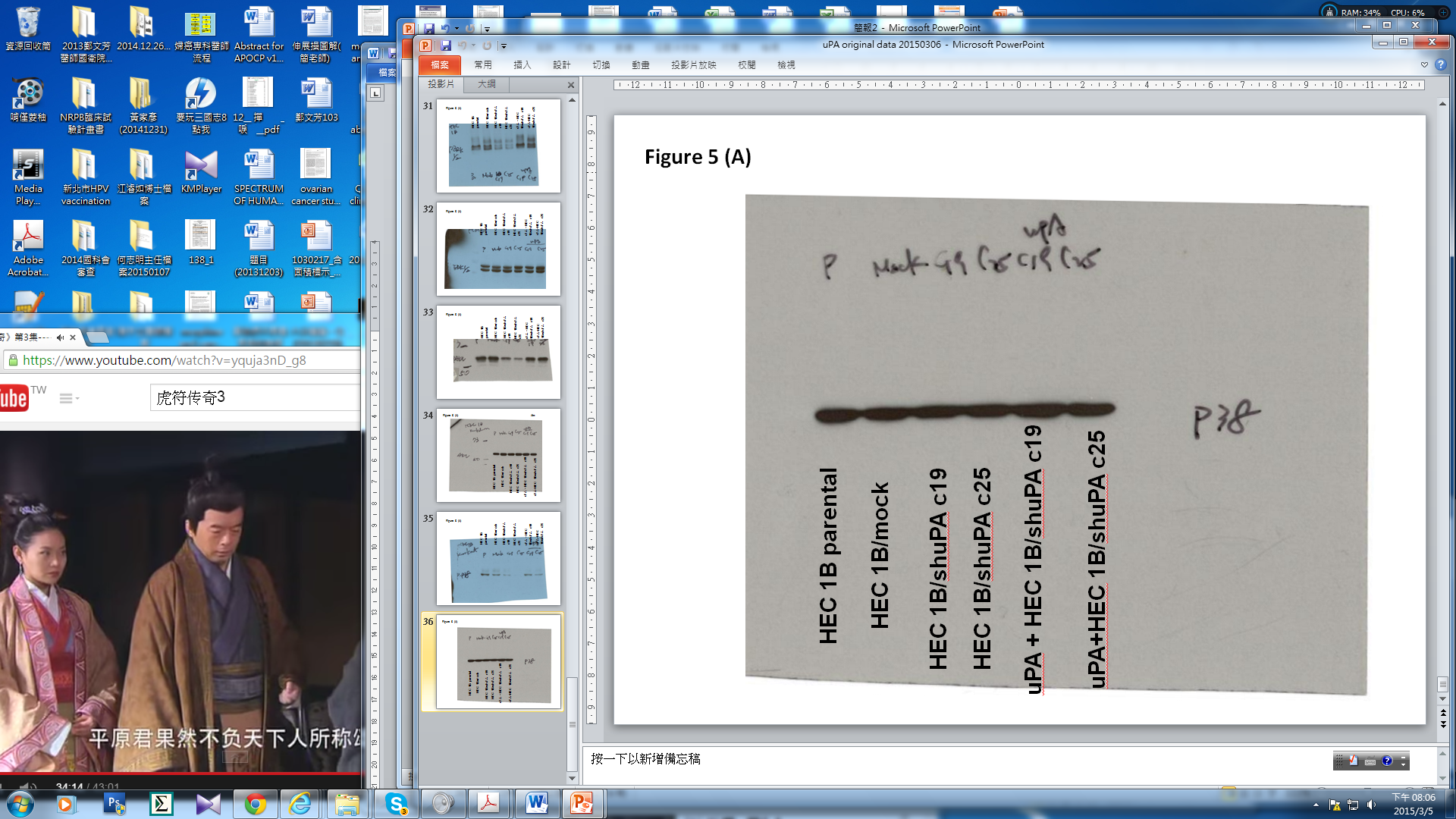

Supplement: Supporting Information [file srep10680-s1.doc]
